# PCAN: phenotype consensus analysis to support disease-gene association

**DOI:** 10.1186/s12859-016-1401-2

**Published:** 2016-12-07

**Authors:** Patrice Godard, Matthew Page

**Affiliations:** 1 Clarivate Analytics (formerly the IP & Science business of Thomson Reuters), 5901 Priestly Dr., #200, Carlsbad, CA 92008 USA; 2 Translational Bioinformatics, UCB Pharma, 208 Bath Road, Slough, SL1 3WE UK

**Keywords:** Disease-gene association, Phenotype, Semantic similarity, Biological networks, Genetics

## Abstract

**Background:**

Bridging genotype and phenotype is a fundamental biomedical challenge that underlies more effective target discovery and patient-tailored therapy. Approaches that can flexibly and intuitively, integrate known gene-phenotype associations in the context of molecular signaling networks are vital to effectively prioritize and biologically interpret genes underlying disease traits of interest.

**Results:**

We describe Phenotype Consensus Analysis (PCAN); a method to assess the consensus semantic similarity of phenotypes in a candidate gene’s signaling neighborhood. We demonstrate that significant phenotype consensus (*p* < 0.05) is observable for ~67% of 4,549 OMIM disease-gene associations, using a combination of high quality String interactions + Metabase pathways and use Joubert Syndrome to demonstrate the ease with which a significant result can be interrogated to highlight discriminatory traits linked to mechanistically related genes.

**Conclusions:**

We advocate phenotype consensus as an intuitive and versatile method to aid disease-gene association, which naturally lends itself to the mechanistic deconvolution of diverse phenotypes. We provide PCAN to the community as an R package (http://bioconductor.org/packages/PCAN/) to allow flexible configuration, extension and standalone use or integration to supplement existing gene prioritization workflows.

**Electronic supplementary material:**

The online version of this article (doi:10.1186/s12859-016-1401-2) contains supplementary material, which is available to authorized users.

## Background

One of the most fundamental questions in biology and biomedical research is how genotype gives rise to phenotype. Unravelling the heterogeneous molecular mechanisms that cause disease will provide the blueprint for practicing precision medicine [1]. Rare human diseases, that exhibit Mendelian inheritance, provide strong links between genotype and phenotype and although individually they affect only a small fraction of the global population, together there are over 3,600 different rare Mendelian diseases associated with over 3,100 different genes. Therefore, a holistic view of existing Mendelian disease genes will not only aid the discovery of novel disorders and associated genes but together resolve fundamental molecular mechanisms that give rise to human phenotypic traits of broad therapeutic potential [2, 3].

Trio whole exome sequencing (WES) has become a routine tool in clinical genetics centers [4, 5] and public research institutes [6] to fuel the rapid discovery of novel Mendelian disease genes. However, even after excluding variants under different models of inheritance from the parents, there will remain a variable number of potential disease causing variants that must be carefully evaluated in order to arrive at a diagnosis. Numerous criteria are considered when prioritizing causal variants, including: control population frequency; predicted pathogenicity [7] and gene-level measures of mutational intolerance [8]. Nevertheless, the final diagnostic coup de grace often comes down to whether other variants in the same gene are known to cause a similar phenotype.

Development of the Human Phenotype Ontology and its systematic use to describe known Mendelian diseases [9] has enabled the automatic quantification of semantic similarity between phenotypes to help diagnose diseases [10] and prioritize disease genes. Similarly, CSI-OMIM [11] allows enhanced querying of rare disease phenotypes by ontological tagging and thematic clustering of phenotype phrases through natural language processing of OMIM Clinical Synopsis entries. Regardless of the approach, if there are no mutations in a candidate gene already known to cause rare disease, then direct phenotype-based approaches are of little use.

A gene’s biological function is the expression of a highly coordinated sequence of interactions between the gene product and other molecules that co-operate as a functional module. Consideration of the signaling environment of a candidate gene can extend the scope of phenotype-based methods to prioritize novel disease genes. ExomeWalker is an indirect phenotype-based method that prioritizes genes based on their network proximity, in the human interactome, to genes that cause similar diseases using a random walk [12]. More recently, indirect phenotype-based approaches have been extended to consider multiple biomedical domain ontologies [13] and incorporated, together with a broad range of variant-level and gene-level properties, into integrated variant prioritization pipelines [14] as shown in Table 1.

**Table 1:** Comparison of PCAN to related methods

Software	Application	Approach	Description	Availability
PCAN	Gene- phenotype exploration	Indirect, phenotype-based	Implements a readily interpreted, statistical definition of phenotype consensus for configurable lists of mechanistically-related genes. Can be used for gene-prioritisation and also versatile, trait-level exploration of gene-phenotype relationships within pathways and biological networks.	R package
CSI-OMIM [11]	Disease diagnosis	Direct, phenotype-based	Improved phenotype searching of NLP processed OMIM Clinical Synopsis descriptions. Phrases are tagged with ontological terms (MESH, UMLS) and clustered into groups of synonymous expressions.	Website
Phenomizer [10]	Disease diagnosis	Direct, phenotype-based	Improved phenotype searching using semantic similarity methods based on HPO annotations for rare diseases.	Website
PhenoDigm [29]	Disease-gene prioritisation	Direct, phenotype-based	Gene prioritisation based on phenotype comparison across model organisms. Model organism trait ontologies (e.g. HPO and MPO) are cross-linked and semantic similarity is computed using the OWLSim algorithm.	Website
Exomewalker [12]	Disease-gene prioritisation	Indirect, phenotype-based	Performs a random walk of the STRING protein-interaction network, seeded with genes linked to diseases with a high semantic similarity to the disorder under investigation. Genes are prioritised based on the random walk score and variant-level criteria combined using a linear model.	Website and command line (via Exomiser)
Syndrome to Gene [32]	Disease-gene prioritisation	Indirect, ontology-based	Use CSI-OMIM to identify genes that cause similar diseases. Quantify gene-relatedness by comparing information vectors derived from 18 source databases using a Jaccard similarity coefficient. Genes are prioritised if they are related to genes that cause similar phenotypes.	Website
OVA [13]	Variant prioritisation	Indirect, ontology-based	Generates extensive, gene-level, multi-ontology annotation profiles for candidate variants and a query phenotype. Direct gene annotations are supplemented with inferred annotations from model organism orthologues and network neighbours. Annotation vectors are compared by computing domain-specific semantic similarities and combined using a Random Forest model to rank variants.	Website
Exomiser [14]	Variant prioritisation	Pipeline	Variant ranking is based on both variant-level properties (allele frequency, pathogenicity) and gene-level semantic similarities for directly linked human diseases, model organism phenotypes as well as network proximity to similar phenotypes using ExomeWalker.	Website and command line

Pleiotropic genes may act at the nexus of different functional modules, so that it is important when evaluating a candidate Mendelian disease gene, to be able to intuitively relate certain traits to different network neighbors or pathways; so called edgotypes [15]. Network exploration of rare disease traits may help design functional validation experiments and identify therapeutic points of intervention in the pathogenic process. Furthermore, methods such as ExomeWalker are developed and optimized specifically for prioritizing gene variants causing Mendelian disorders. They are not easily customized to address the same kind of issue for model organisms, consider datasets that speak to different genetic architectures such as Genome Wide Association Studies (GWAS), or allow flexible definition of related genes sets (e.g. gene family members).

Here we report Phenotype Consensus Analysis (PCAN); an indirect phenotype-based method that quantifies the consensus similarity of genetic disorders linked to the mechanism of a putative disease causing gene. PCAN makes use of widely adopted knowledge resources for protein-protein interactions (STRING [16]) and signaling pathways (Reactome [17]) and the comprehensive HPO annotation resource [9]. Our approach allows support for the discovery of novel disease genes and naturally lends itself to the mechanistic deconvolution of diverse phenotypes. We validate our method using all existing rare diseases of known genetic etiology present in OMIM. We provide PCAN to the community as an R package, available to download from Bioconductor, to allow integration into existing rare disease variant prioritization workflows and support extensive customization and versatile exploration of the molecular etiology of disease.

## Implementation

### PCAN workflow

**Fig. 1:**
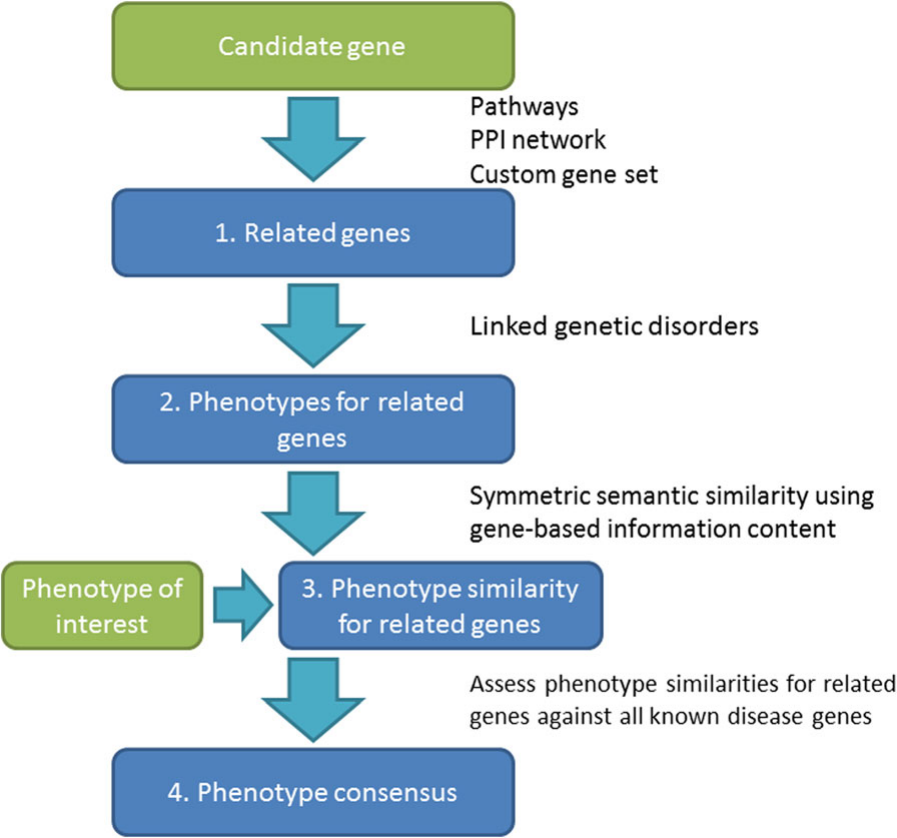
PCAN workflow. The typical PCAN workflow followed to assess the relationship between a candidate gene and a disease of interest based on genes mechanistically related to the candidate (from pathways or protein-protein interaction networks) and the Mendelian disorders they are known cause. *Green boxes* indicate user provided inputs to the method

Here we present the PCAN workflow which can be applied to assess how likely it is that mutation of a candidate gene causes a particular disease phenotype under investigation (Fig. 1). Firstly, genes that are mechanistically related to the gene candidate are identified using a reference set of canonical pathways or a protein-protein interaction network (step 1). PCAN also allows specification of custom related gene sets or flexible extension of existing gene sets. Each gene in the related gene set is linked to phenotypic traits that describe the Mendelian diseases they are known to cause (step 2). If a gene in the related gene set is not known to cause human Mendelian disease, it is not considered further in the analysis. For each remaining gene, semantic similarity is computed between gene-linked phenotypes and the phenotype of interest (step 3). Finally a one-sided Mann-Whitney U test is applied to determine if members of the related gene set demonstrate a consensus phenotype with respect to the disease under analysis (step 4). If there are multiple candidate genes, the workflow can be repeated for each gene.

### Prior knowledge resources (step 1 and 2)

**Fig. 2:**
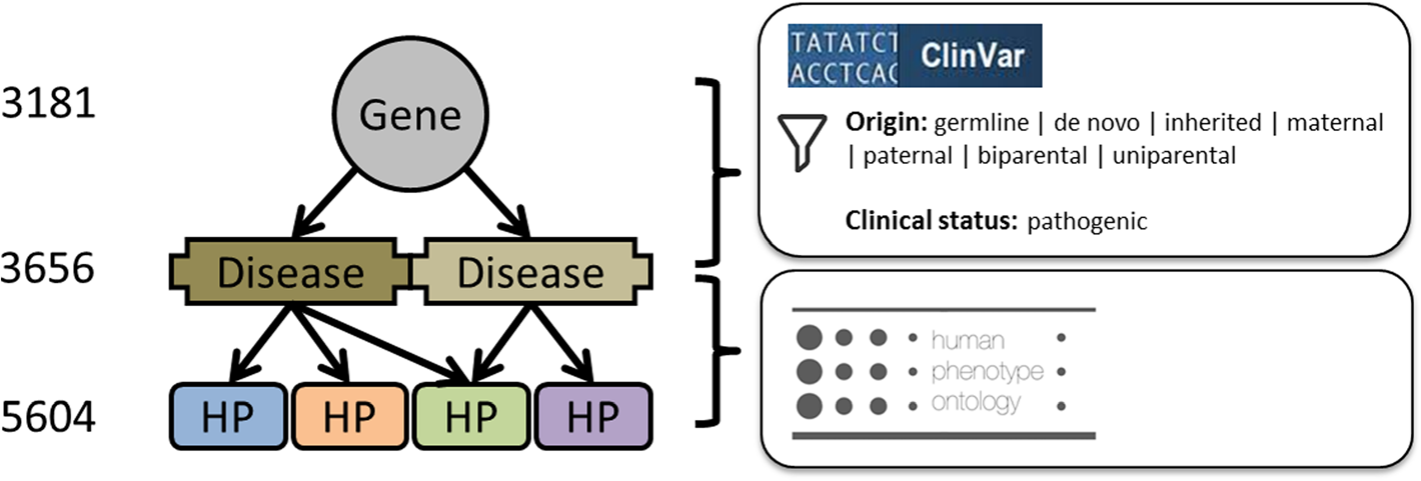
PCAN prior knowledge resources. Public resources used to link genes to phenotypic abnormalities based on the genetic diseases each gene causes. The HPO phenotype annotation resource (build #1039) was used to link HP terms to OMIM disorders and ClinVar (version of May 2015) was used to retrieve genes that cause OMIM disorders. Total counts of each distinct entity type in the resultant gene-trait resource are provided

Two resources were used to enable genes to be linked to phenotypic abnormalities based on the clinical symptomatology of the genetic disorders each gene is known to cause (Fig. 2).

The Human Phenotype Ontology (HPO) [9] (build #1529) is used to formally describe phenotypes, as sets of human phenotype (HP) terms, to enable their intercomparison. We only consider HP terms descended from the “Phenotypic abnormality” (HP:0000118) branch of the HPO. The phenotype annotation resource (build #1039) provided by the HPO was used to list HP terms assigned to each OMIM disorder.

The ClinVar database [18] (version of May 2015) was used to identify genes (using Entrez Gene [19] identifiers) causally linked to Mendelian diseases. Here we focused on diseases reported within OMIM and linked variants with a pathogenic clinical status and one of the following origins: germline, de novo, inherited, maternal, paternal, biparental or uniparental. In summary 3,181 human genes were associated to 3,656 diseases to which at least one HP term descendant of “Phenotypic abnormality” is related (4,549 associations in total).

Pathway and biological network resources were used to identify mechanistically related genes in order to assess phenotype consensus. Two pathway resources were used to identify genes that encode proteins, which function in common signaling pathways: Reactome and Thomson-Reuters’ Metabase. Reactome [17] is a free, open-source, curated and peer reviewed pathway database. Here we used version v52 to associate 7,580 human genes to 1,345 individual pathways. Metabase (http://thomsonreuters.com/en/products-services/pharma-life-sciences/pharmaceutical-research/metabase.html) is a comprehensive manually curated database of mammalian biology and medicinal chemistry data. Here we used version 6.20.66604, which includes 6,978 human genes within 1,465 pathways.

To identify gene neighbors, two biological network databases were used: the STRING database and once again Metabase. STRING [16] is a database of known and computationally predicted protein interactions. Interactions include both direct (physical) and indirect (functional) associations. We focused on the 1,249,080 direct interactions involving 17,114 human genes, within STRING version 10. STRING also provides a measure of confidence for each interaction as a score ranging from 0 to 1,000. In the following analyses we consider either the whole STRING network or only a high quality (HQ) subnetwork involving interactions with a score [20] greater than or equal to 0.5 (507,298 interactions between 13,712 genes). Additionally, 862,660 interactions, involving 23,136 genes, were extracted from Metabase. Among these interactions, 238,171 (involving 17,265 genes) are assigned a high trust and form the Metabase high quality (HQ) subnetwork.

### Phenotype comparison using semantic similarity (step 3)

The semantic similarity between two HP terms was computed as described by Köhler et al. [10]. First an information content (IC) was computed for each HP term as a measure of its specificity:$I{C}_p=- \ln \left(\frac{\left|p\right|}{\left|\Omega \right|}\right)$ where |*p*| is the number of genes directly associated to the HP term or one of its descendants [21] and |Ω| is the total number of genes linked to Mendelian diseases.

Then we used the Resnik method to measure the semantic similarity between two HP terms t1 and t2 (*SS*_*t*1,*t*2_) as the IC of the most informative common ancestor (MICA) [22].

We finally use the same symmetric similarity measure used by Köhler et al. [10] to compare two sets of HP terms corresponding to two disease phenotypes:

$$ sim\left(Q\to D\right)=\frac{{\displaystyle {\sum}_{t1\in Q}}\underset{t2\in D}{ \max }S{S}_{t1,t2}}{\left|Q\right|} $$

$$ si{m}_{symmetric}\left(D,Q\right)=\frac{sim\left(D\to Q\right)+sim\left(Q\to D\right)}{2} $$

Briefly, for each query HP term (Q) the best match among disease HP terms (D) is identified and the average of the best match scores for all the query terms is computed. The same calculus is applied with disease terms compared to query terms (*sim*(*D* → *Q*)). The symmetric semantic similarity is the average of these two scores.

### Computing phenotype consensus (step 4)

The aim of our method is to compare a phenotype of interest with Mendelian diseases caused by a set of genes mechanistically related to a candidate causal gene. Candidate-related genes can belong to the same pathway (here from Reactome or Thomson-Reuters’ Metabase) or encode neighbors in a molecular network (here from STRING or Thomson-Reuters’ Metabase). Each knowledge resource for identifying candidate-related genes can be considered a different approach for sampling molecular mechanism. If genes in a mechanism sub-sample tend to have a greater semantic similarity than all disease-associated genes, the candidate gene is assigned a significant phenotype consensus. Specifically, the symmetric semantic similarity of the query HP terms is computed for all the genes with at least one linked HP term. Finally, a one-sided Mann-Whitney U test is applied to determine whether the symmetric semantic similarities for candidate-related genes are significantly greater than values for all other disease-associated genes.

### Software availability

The method is implemented in a Bioconductor [23] package (http://bioconductor.org/packages/PCAN/).

## Results

### Rationale and workflow

**Fig. 3:**
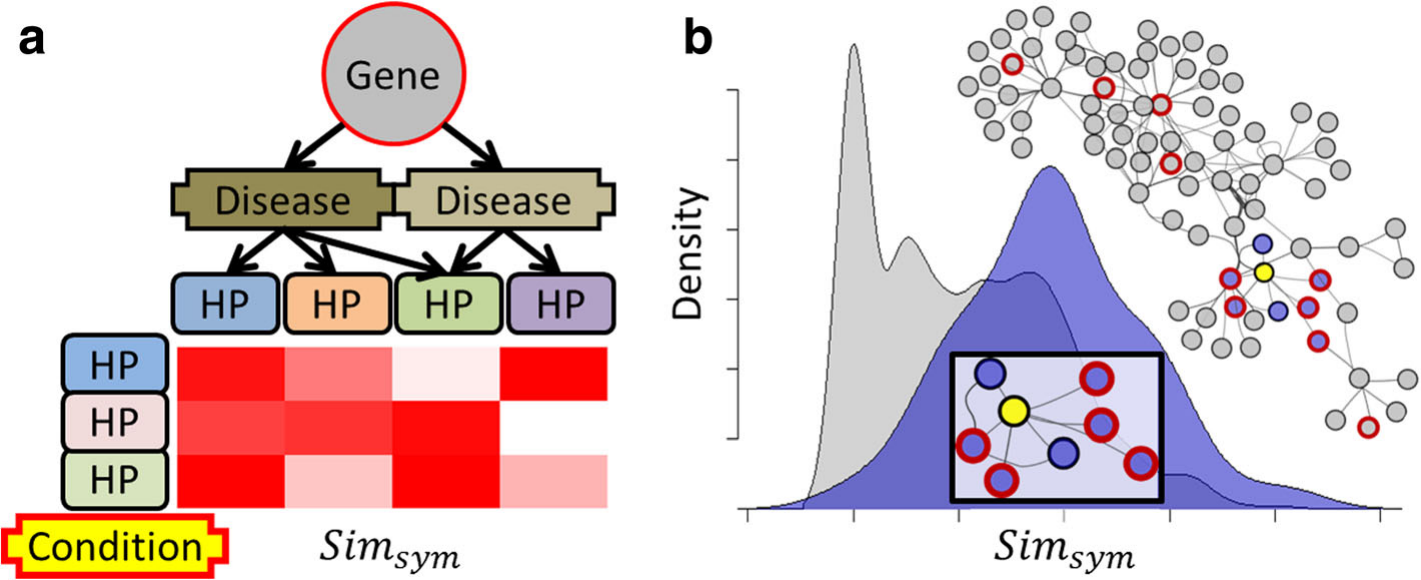
Assessing a gene's relevance for a condition by applying a pathway consensus approach. **a** Each gene, which is known to be involved in at least one genetic disorder, is associated to the corresponding HP terms. These HP terms are compared to those related to the disease of interest by computing a symmetric semantic similarity score. **b** The scores of all genes related to the gene of interest are compared to scores for all known Mendelian disease genes. Here the gene candidate is in *yellow* and its direct neighbors are in *blue*. Nodes surrounded in *red* correspond to genes with a high semantic similarity score for the disease under focus

The aim of the PCAN method is to assess the likelihood that a gene will cause an observed set of phenotypes, by quantifying the consensus phenotype similarity to described disorders in the gene’s signaling neighborhood. To achieve this goal, first all Mendelian disease genes are annotated with standardized trait labels (HP terms) from the Human Phenotype Ontology (HPO) [9] according to the genetic disease or diseases they cause. Gene-linked HP terms are compared to HP terms that describe the query phenotype by computing a symmetric semantic similarity score. Different knowledge resources such as biological pathways or protein association networks are used to identify genes mechanistically related to the candidate gene. Finally a one-sided Mann-Whitney U test is applied to determine if the symmetric semantic similarity scores of genes that are part of a related molecular mechanism tend to be greater than all other genes annotated with HP terms (Fig. 3).

**Fig. 4:**
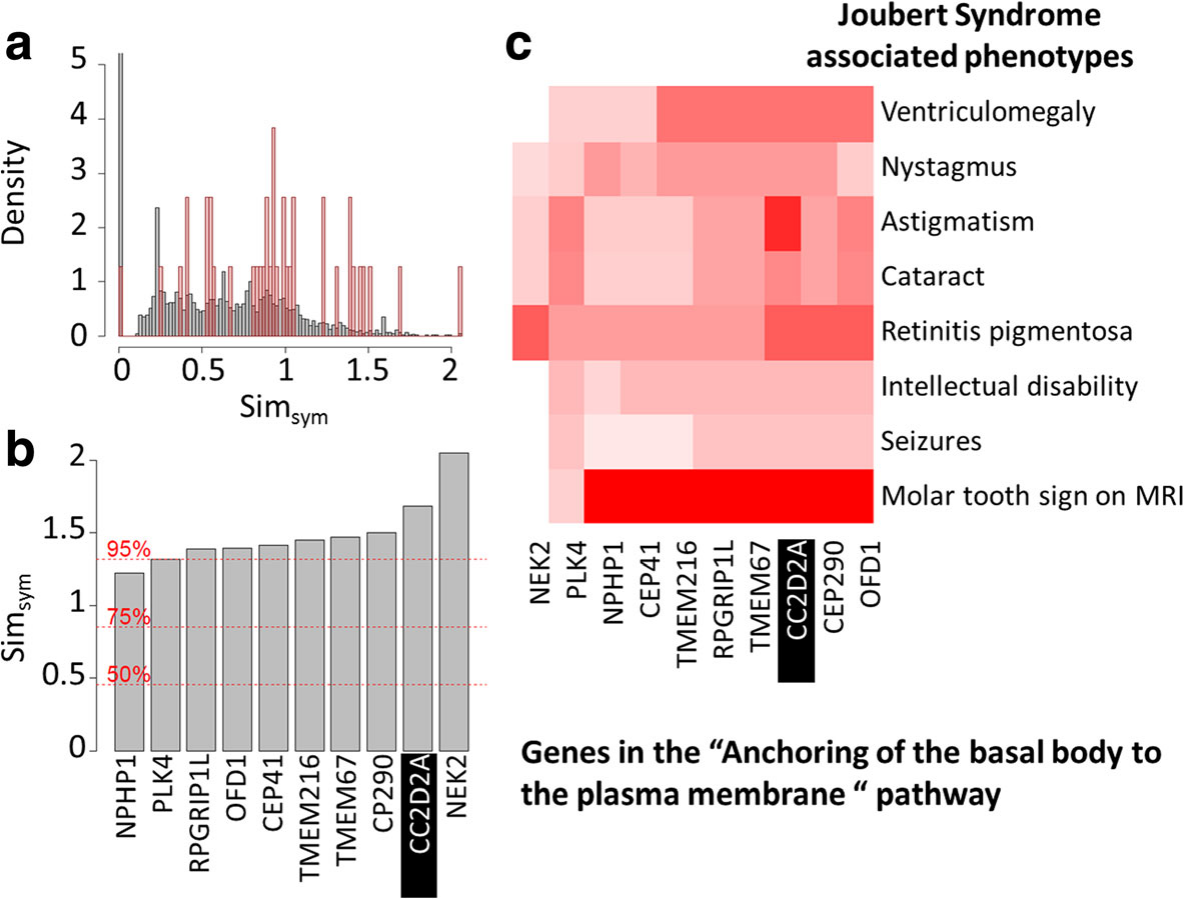
Comparing the genes belonging to the “Anchoring of the basal body to the plasma membrane” pathway to the HP terms related to Joubert syndrome. **a** Distribution of symmetric semantic similarity scores of genes for the 8 HP terms related to Joubert syndrome. The *red bars* correspond to the distribution of the scores of genes belonging to the pathway of interest. The *grey bars* correspond to the distribution of the scores for all the other genes. (The density of scores equal to 0 is truncated; its actual value is 12.8) **b** Symmetric semantic similarity scores of genes belonging to the pathway of interest. The gene candidate, CC2D2A, is highlighted. *Dashed red lines* show the value of three specific quantiles: 50, 75 and 95%. **c** Heatmap showing the best semantic similarity between each gene in the pathway of interest (columns) and each HP term under focus (rows). The *red* intensity of each square corresponds to the highest semantic similarity score between the HP term of interest and the gene associated HP terms (*white*: 0 and *red*: 5.2). The gene candidate, CC2D2A, is highlighted. In figures (**b**) and (**c**), only the top 10 genes are shown. Additional file 3: Figure S1 shows results for all the genes in the pathway

PCAN allows the user to prioritize putative disease causing genes but importantly, it also enables granular, biological interpretation of the output to relate high-scoring, matching traits to specific sub-processes and interactions. To illustrate this we took Joubert syndrome [24] as an example. Joubert syndrome is a genetically heterogeneous group of disorders first described in 1969 and characterized by atrophy of the cerebellar vermis and malformation of the brain stem leading to physical, mental and sometimes visual impairment that can vary in severity. Joubert syndrome 9 [25] was linked to 8 HP terms using the phenotype annotation resource of the HPO: “Astigmatism” (HP:0000483), “Retinitis pigmentosa” (HP:0000510), “Cataract” (HP:0000518), “Nystagmus” (HP:0000639), “Intellectual disability” (HP:0001249), “Seizures” (HP:0001259), “Ventriculomegaly” (HP:0002119) and “Molar tooth sign on MRI” (HP:0002419). Joubert syndrome 9 describes a genetically defined subset of Joubert syndrome caused by different recessive mutations in CC2D2A [26–28]. CC2D2A encodes a coiled-coil and calcium domain binding protein that belongs to the “Anchoring of the basal body to the plasma membrane” Reactome [17] pathway; a process involved in the assembly of the primary cilium. Among the 88 genes belonging to this pathway, 39 were associated to at least one genetic disease and could therefore be associated to at least one HP term. Figure 4a shows that the symmetric semantic similarity scores of these genes are, as a population, significantly higher than the scores of all remaining 3143 disease-associated genes (*p*-value < 10^−8^). Figure 4b and c illustrate how genes belonging to the same pathway and hence mechanism as CC2D2A cause similar diseases to Joubert syndrome by showing both their symmetric semantic similarity scores and detailing the contributing query HP term best matches. 8 of the 39 pathway genes have a score higher than 95% of all the genes for which a score could be calculated and correspond to genes in the same OMIM Phenotypic Series. As expected, the majority of these genes show consistently high similarity for many of the HP terms of interest, especially “Molar tooth sign on MRI”; one of the defining hallmarks of Joubert syndrome [24].

### Validation and performance

Our method is founded on the assumption that mechanistically related genes will cause diseases that have similar traits; our concept of phenotype consensus. To test this assumption, we exhaustively attempt to rediscover known rare disease genes within OMIM, when the existing gene-disease link and therefore HP term annotations for the true causal gene have been specifically removed from our reference data. We apply the PCAN method for each of the 4,549 known gene-disease associations, in each case removing HP annotations for the true causal gene. We assess whether PCAN assigns a significant phenotype consensus to the true causal genes and simultaneously apply the same approach to 100 randomly selected genes among those not associated to the disease of interest to enable us to evaluate specificity.

The performance of the method was assessed using three different measures. The *potential* of the method indicates the proportion of gene-disease associations for which a score can be computed. If there are no known interaction partners of a gene of interest, then the PCAN method cannot be applied. The *area under the Receiver Operating Characteristic (ROC) curve* (AUC) measures the accuracy of the method. Finally the *average rank* of the candidate among the 100 negative control genes assesses the efficacy of the method.

This validation scheme and the different derived measures of performance enabled us to evaluate different configurations of the PCAN method concerning approaches to sample mechanistically related genes. Genes mechanistically related to the gene candidate were identified using different protein interaction and pathway resources. Both Reactome and Thomson-Reuters’ Metabase were used to identify genes belonging to the same pathway(s) as the candidate gene. When the gene candidate belongs to several pathways we report the result for the pathway most significantly associated to the query HP terms. The STRING database and Metabase were used to find interaction neighbors for the candidate gene of interest. Both networks contain directional interactions, so we were able to consider either all interaction neighbors or only downstream or upstream sub-sets. We also filtered the neighbors according to the quality of the relationship as captured by the alternative network resources.

Table 2 compares the performance of the different options (ROC curves are provided in Additional file 1: Figure S2). When using the whole network from Metabase, the phenotype consensus method could provide a score for 96% of the cases (potential = 96%). However the corresponding AUC is only 68% with the correct candidate found in the top 22% of the analyzed genes (median occurrence). In contrast, using only upstream neighbors in the high quality (HQ) STRING network produces an AUC of 75% with a higher median occurrence of 14% of the analyzed genes. However, the potential of the method with such on option is only of 40%. In general, accuracy of the method increases when using a HQ network whereas its potential decreases.

**Table 2:** Performance of the pathway consensus approach depending on the prior knowledge used to identify genes related to the candidate under focus

Type of knowledge	Resource	Number of results	Potential	AUC	Median rank
Pathways	MetaBase	2355	52%	74%	19%
Pathways	Reactome	2669	59%	73%	20%
Neighbors	Metabase	4367	96%	68%	22%
Neighbors	MetaBase HQ^a^	3623	80%	73%	16%
Neighbors	STRING	3705	81%	71%	19%
Neighbors	STRING HQ^a^	3247	71%	74%	14%
Upstream neighbors	Metabase	4362	96%	65%	27%
Upstream neighbors	MetaBase HQ^a^	3399	75%	70%	20%
Upstream neighbors	STRING	2158	47%	73%	16%
Upstream neighbors	STRING HQ^a^	1825	40%	75%	14%
Downstream neighbors	Metabase	3352	74%	74%	16%
Downstream neighbors	MetaBase HQ^a^	2600	57%	74%	15%
Downstream neighbors	STRING	2069	45%	73%	18%
Downstream neighbors	STRING HQ^a^	1722	38%	74%	15%
Pathways + Neighbors	Metabase + MetaBase HQ^a^	3746	82%	75%	15%
Pathways + Neighbors	Reactome + MetaBase HQ^a^	3861	85%	75%	16%
Pathways + Neighbors	MetaBase + STRING HQ^a^	3515	77%	76%	14%
Pathways + Neighbors	Reactome + STRING HQ^a^	3617	80%	76%	15%

^a^HQ corresponds to high quality network as described in material and methods

This led us to assess the performance of combined results from the PCAN method configured to use different network neighbor and pathway knowledge resources. Different combinations of HQ network and pathway were considered and summarization simply involved taking the lowest *p*-value from either PCAN alternative. As shown in Table 2, such combinations produce an improvement both in terms of potential and accuracy. For example, a score can be computed for 77% of the cases when combining pathways from Metabase and neighbors from the HQ STRING network. Furthermore this combination is performant, with an AUC of 76% and a median rank of 14% of the analyzed genes.

Finally, to further assess the ability of PCAN to predict Mendelian diseases in the future, we extended our analysis to consider 759 novel genetics findings published in clinVar between May 2015 and August 2016, applying an identical validation procedure with the same prior knowledge resources. Observed AUCs for the newly reported genetic findings tend to be lower than their equivalent measures from the retrospective validation (obtained with clinVar from 2015) for the different pathway and network resources considered. This emphasizes the sensitivity of the method to the current level of biological understanding of genetic disorders and the importance of being able to flexibly explore different related gene sets from numerous sources (Additional file 2: Table S1).

Nevertheless, the results from this retrospective validation do support the underlying assumption that mechanistically related genes produce similar diseases when their function is impacted. Therefore, PCAN will help quantify the biological relevance of candidate genes to the observed phenotype in a way that will improve decision making and is applicable to the discovery of novel disease genes.

## Discussion

Here we describe PCAN, a novel, indirect phenotype-based method to support the identification of disease genes by evaluating whether similar phenotypes are linked to genes in the same signaling neighborhood. Phenotype consensus can be considered as a proxy for the biological relevance of a candidate gene’s function to an observed phenotype. PCAN exploits the wealth of available prior knowledge from reported Mendelian disease genes and the molecular interactions and biological pathways in which genes are involved. Here we validate the underlying assumption that genetic perturbation of members of the same molecular mechanism produces a consensus phenotype. Specifically, we test if PCAN can accurately rediscover known disease genes when the equivalent gene-disease links are excluded from the method’s reference data. During validation we consider different options for supplying molecular network and pathway information. Combining different sources of molecular interaction information increased both the number of cases that could be evaluated and the accuracy of the results.

Both direct [10, 27] and in-direct [12] phenotype-based methods have been developed to prioritize variants from WES of patients with Mendelian diseases (see Table 1). These approaches all make use of genotype-phenotype links provided by reported human Mendelian diseases. In contrast, PhenoDigm compares human disease phenotypes to mouse genetic models in an analogous direct semantic similarity methodology [29]. This is enabled by cross-referencing the HPO with the equivalent Mammalian Phenotype Ontology [30]. A recently published method termed Ontology Variant Analysis, combines phenotype level annotation sets from numerous biomedical ontologies with network sampling as part of an integrated scoring scheme [13]. However the extensive ontology cross-referencing that forms a fundamental part of this method may unwittingly incur information leakage that is difficult to account for during validation.

PCAN is a gene prioritization method that can also be applied to prioritize variants from WES, using readily configurable and updateable prior knowledge of gene-phenotype links and mechanistically related genes. Here we demonstrate the capabilities of the method using ClinVar and HPO to link genes to phenotypic traits and different network and pathway resources. Within the Bioconductor R package, we provide tools to maintain local, up-to-date copies of ClinVar and HPO that are easy to use with PCAN. The package also facilitates addition of new resources linking genes to phenotype and different phenotype ontologies, for example from model organisms. We use two approaches for sampling the molecular mechanism of a gene: a nearest neighbor network derived from the interactome and curated, canonical pathways. Different pathway resources and any other lists of functionally related genes could be considered, including: community partitions of the human interactome; gene-family members and Gene Ontology (GO) Biological Process sets.

As well as configurability, another key advantage of our tool is the ability to identify phenotype traits linked to neighboring genes, which are contributing highly to the observed phenotype semantic similarity. Ease of deconvolution is of particular importance when considering pleiotropic causal genes where subcategories of traits may relate to particular subsets of interaction partners or a particular signaling axis within a pathway. A firmer grasp on the molecular mechanism underlying the condition will support the definition of experiments to validate and elaborate the causal hypothesis and help identify druggable points of therapeutic intervention.

PCAN is a standalone gene-phenotype exploration tool which can be used in broader contexts than variant prioritization from WES data. For example, the prioritization of genes in the same linkage disequilibrium block as single nucleotide polymorphism (SNP) disease associations from genome wide association studies (GWAS). Each co-segregating gene may harbor the true causal variant, which PCAN can be used to highlight if the signaling environment of the gene is linked to similar diseases. In this context, extension of the HPO annotation to common diseases will be particularly useful [31].

## Conclusions

PCAN is a modular and flexible toolkit to support gene prioritization according to phenotype similarity. It takes advantage of the knowledge related to known molecular partners of the gene candidates. Compared to other available methods, PCAN can be easily customized and used with different knowledge resources. The method supports deconvolution of the final score to provide an intuitive understanding of the results in terms of contributory gene-trait links. We provide PCAN to the community as an R package, available to download from Bioconductor (http://bioconductor.org/packages/PCAN/), to facilitate flexible integration into any genetics analysis pipeline. We feel this is an appropriate compromise between ease of use, methodological transparency and analytical flexibility.

## Additional files

Additional file 1: Figure S2. ROC curves comparing sensitivities and specificities of different pathway consensus approaches. Numbers along the ROC curve indicate representative *p*-value thresholds. Confidence intervals are based on specificity measures. (PDF 67 kb)
Additional file 2: Table S1. Performance of the pathway consensus approach on 759 new genetics findings published in clinVar between May 2015 and August 2016 depending on the prior knowledge used to identify genes related to the candidate gene of interest. HQ corresponds to high quality network as described in material and methods. (XLSX 12 kb)
Additional file 3: Figure S1. Comparison of genes within the “Anchoring of the basal body to the plasma membrane” pathway to HP terms describing Joubert syndrome. (a) Distribution of symmetric semantic similarity scores of genes for the 8 HP terms related to Joubert syndrome. The red bars correspond to the distribution of the scores of genes belonging to the pathway of interest. The grey bars correspond to the distribution of the scores for all the other genes. (The density of scores equal to 0 is truncated; its actual value is 12.8). (b) Symmetric semantic similarity scores of genes belonging to the pathway of interest. The gene candidate, CC2D2A, is highlighted. In the supplementary figure, the solid red line corresponds to the quantiles of the scores of all the genes. Dashed red lines show the value of three specific quantiles: 50, 75 and 95%. (c) Heatmap showing the best semantic similarity between each gene in the pathway of interest (columns) and each HP term under focus (rows). The red intensity of each square corresponds to the highest semantic similarity score between the HP term of interest and the gene associated HP terms (white: 0 and red: 5.2). The gene candidate, CC2D2A, is highlighted. (PPTX 198 kb)

## Abbreviations

AUC: Area under the ROC curve
GWAS: Genome wide association study
HP: Human phenotype
HPO: Human Phenotype Ontology
HQ: High quality
IC: Information content
LoF: Loss-of-function
MICA: Most informative common ancestor
OMIM: Online Mendelian Inheritance in Man
PCAN: Phenotype Consensus Analysis
ROC: Receiver operating characteristic curve
SNP: Single nucleotide polymorphism
STRING: Search tool for the retrieval of interacting genes/proteins
WES: Whole exome sequencing
