# Supplementary material for: PCAN: phenotype consensus analysis to support disease-gene association
Source: BMC Bioinformatics. 2016 Dec 7;17:518. doi: 10.1186/s12859-016-1401-2 (PMC5142268; doi:10.1186/s12859-016-1401-2)
Supplement: Additional file 1: Figure S2. — ROC curves comparing sensitivities and specificities of different pathway consensus approaches. Numbers along the ROC curve indicate representative p-value thresholds. Confidence intervals are based on specificity measures. (PDF 67 kb) [file 12859_2016_1401_MOESM1_ESM.pdf]

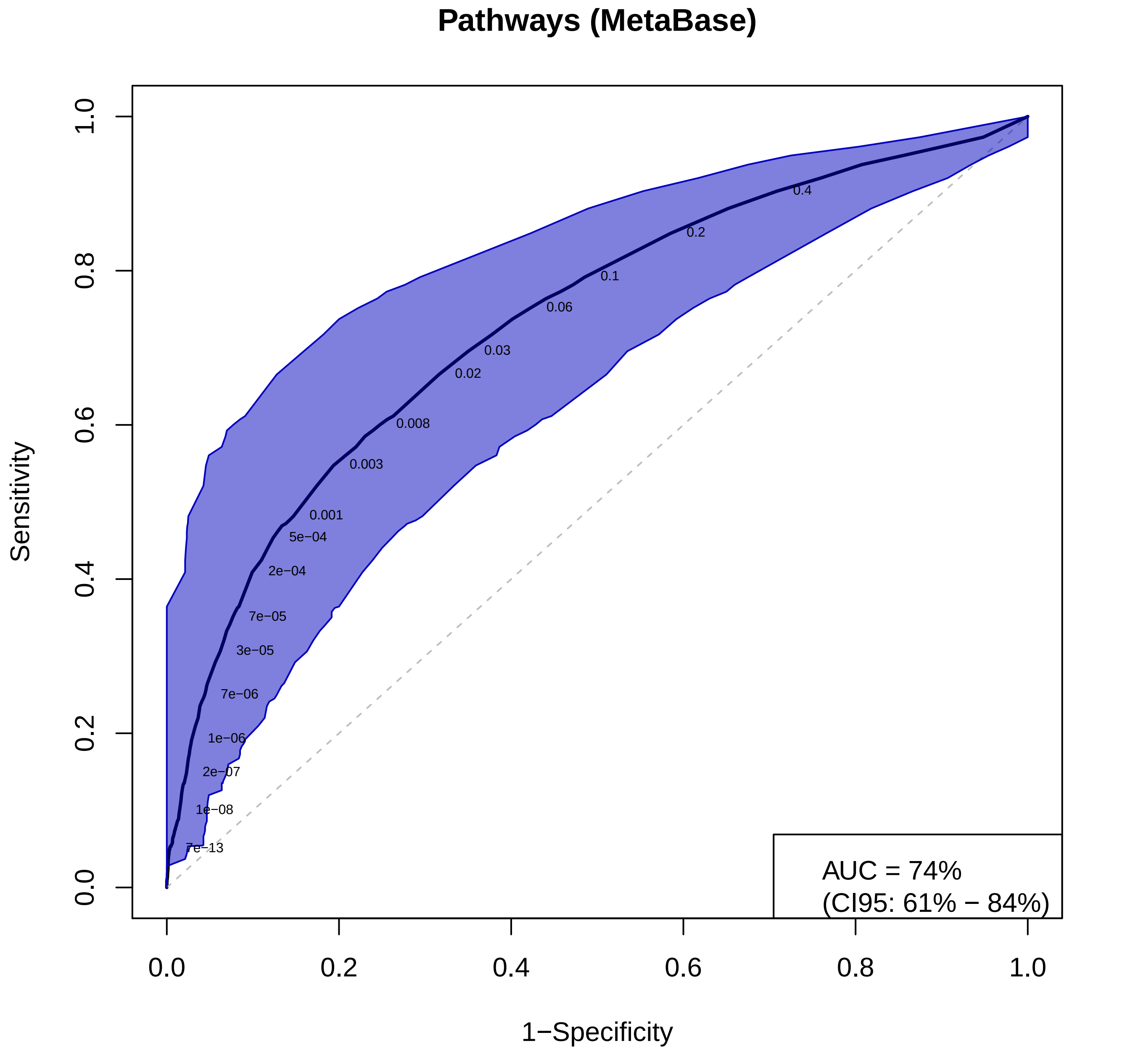
Pathways (MetaBase)
Sensitivity
1−Specificity
0.0
0.2
0.4
0.6
0.8
1.0
0.0
0.2
0.4
0.6
0.8
1.0
0.4
0.2
0.1
0.06
0.03
0.02
0.008
0.003
0.001
5e−04
2e−04
7e−05
3e−05
7e−06
1e−06
2e−07
1e−08
7e−13
AUC = 74%
(CI95: 61% − 84%)

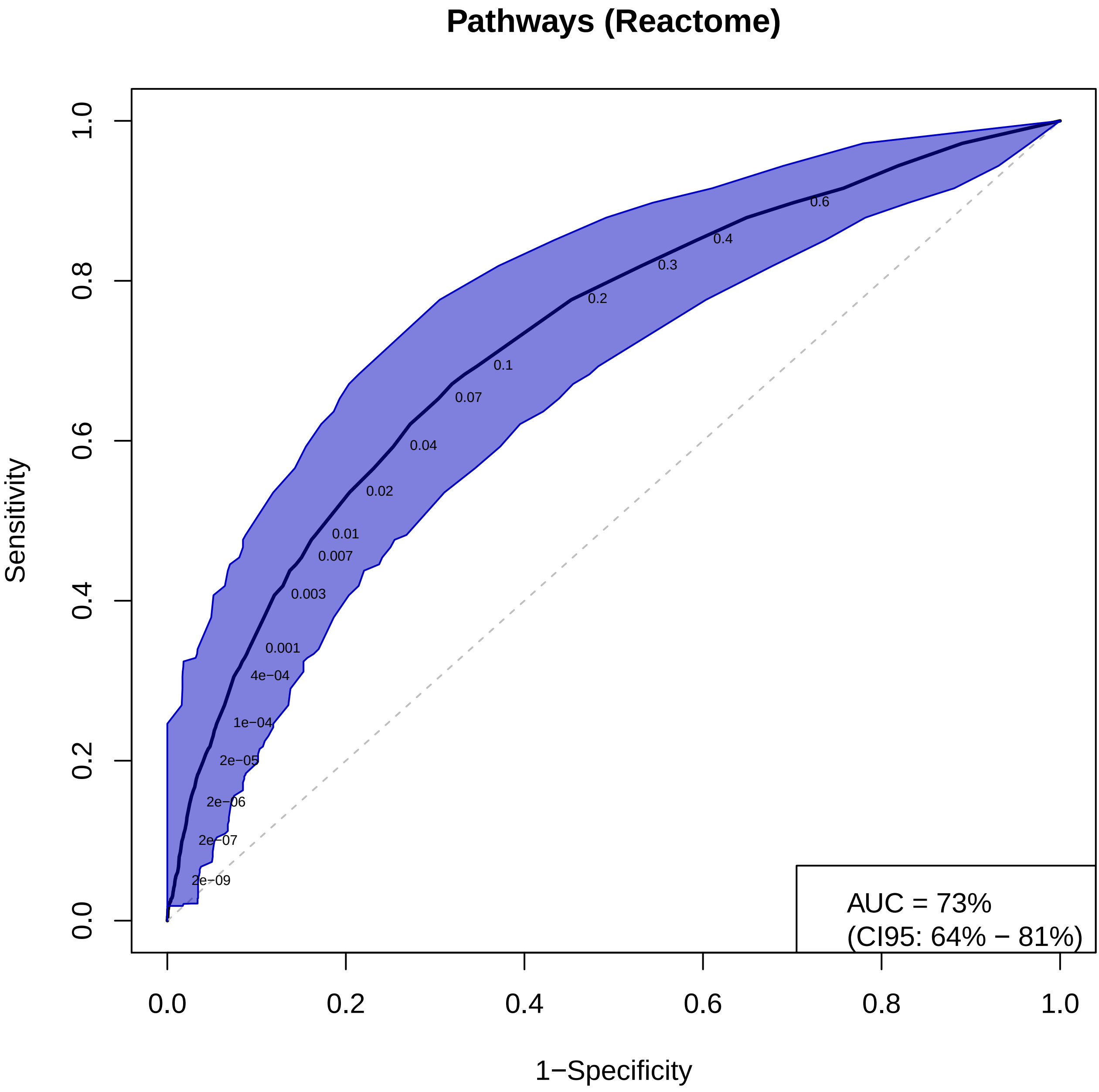

Pathways (Reactome)
Sensitivity
1−Specificity
0.0
0.2
0.4
0.6
0.8
1.0
2e−09
2e−07
2e−06
2e−05
1e−04
4e−04
0.001
0.003
0.007
0.01
0.02
0.04
0.07
0.1
0.2
0.3
0.4
0.6
AUC = 73%
(CI95: 64% − 81%)

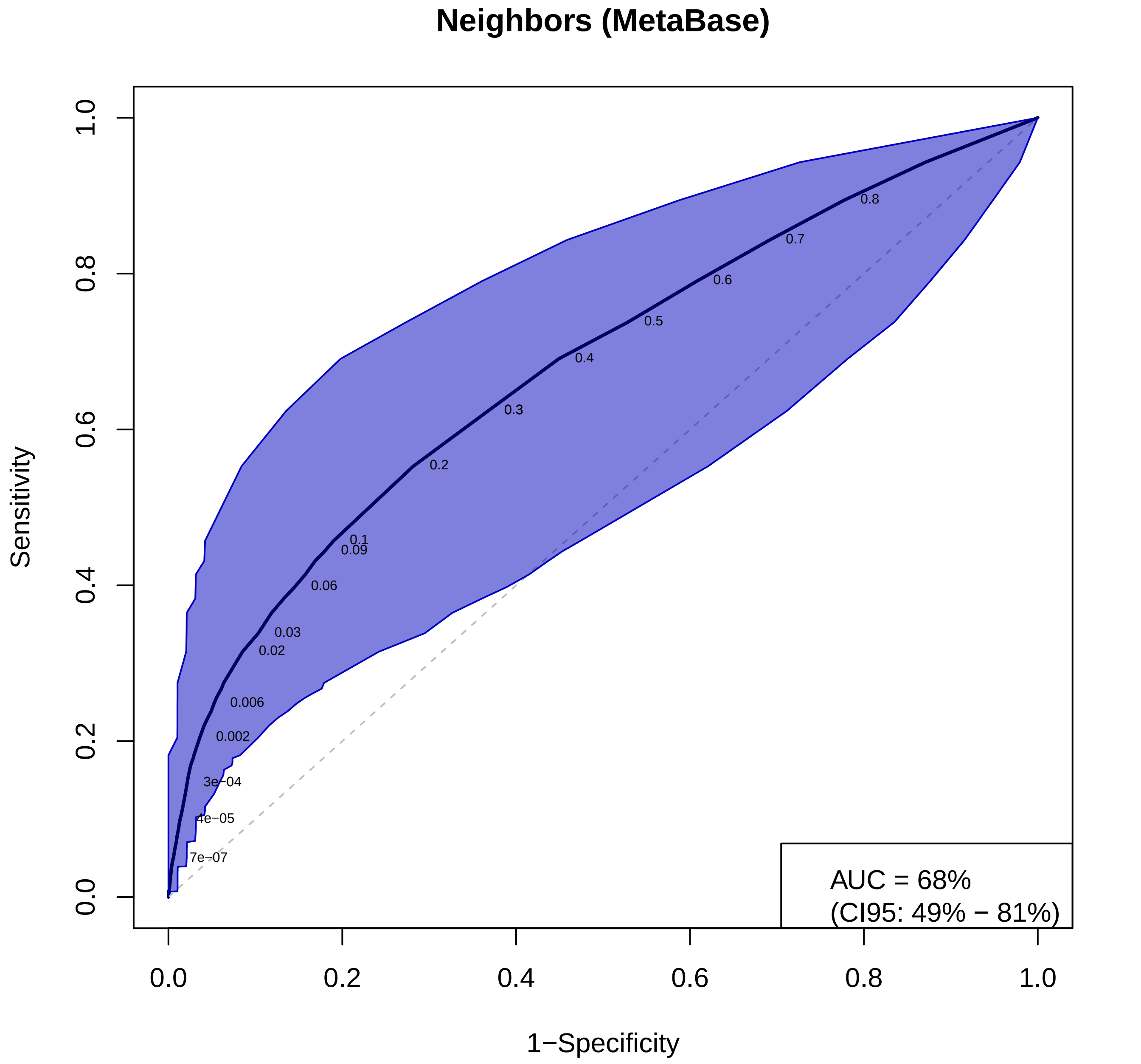

Neighbors (MetaBase)
Sensitivity
1−Specificity
0.0
0.2
0.4
0.6
0.8
1.0
7e−07
4e−05
3e−04
0.002
0.006
0.02
0.03
0.06
0.09
0.1
0.2
0.3
0.4
0.5
0.6
0.7
0.8
AUC = 68%
(CI95: 49% − 81%)

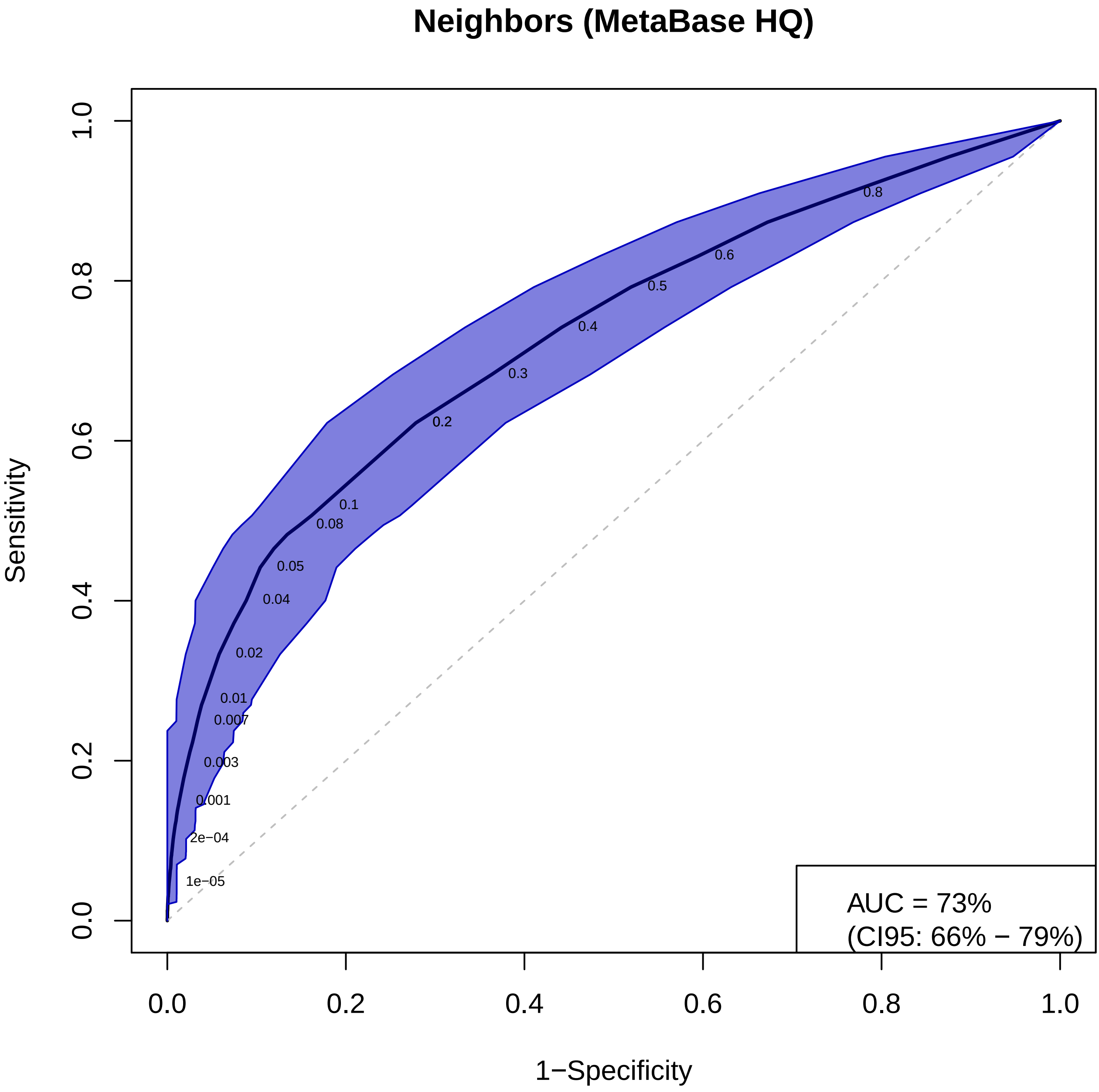

Neighbors (MetaBase HQ)
Sensitivity
1−Specificity
0.0
0.2
0.4
0.6
0.8
1.0
1e−05
2e−04
0.001
0.003
0.007
0.01
0.02
0.04
0.05
0.08
0.1
0.2
0.3
0.4
0.5
0.6
0.8
AUC = 73%
(CI95: 66% − 79%)

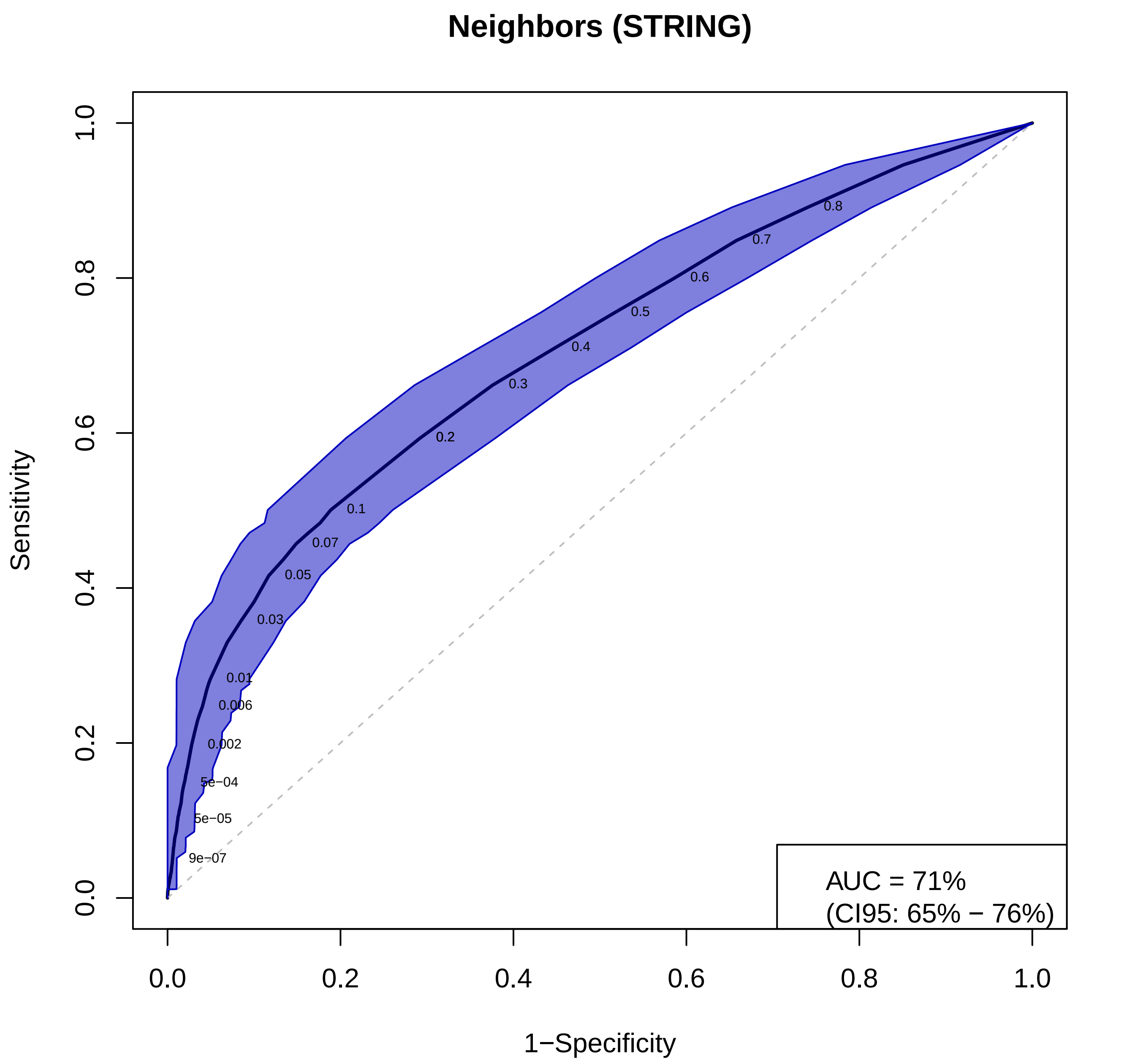

Neighbors (STRING)
Sensitivity
1−Specificity
0.0
0.2
0.4
0.6
0.8
1.0
9e−07
5e−05
5e−04
0.002
0.006
0.01
0.03
0.05
0.07
0.1
0.2
0.3
0.4
0.5
0.6
0.7
0.8
AUC = 71%
(CI95: 65% − 76%)

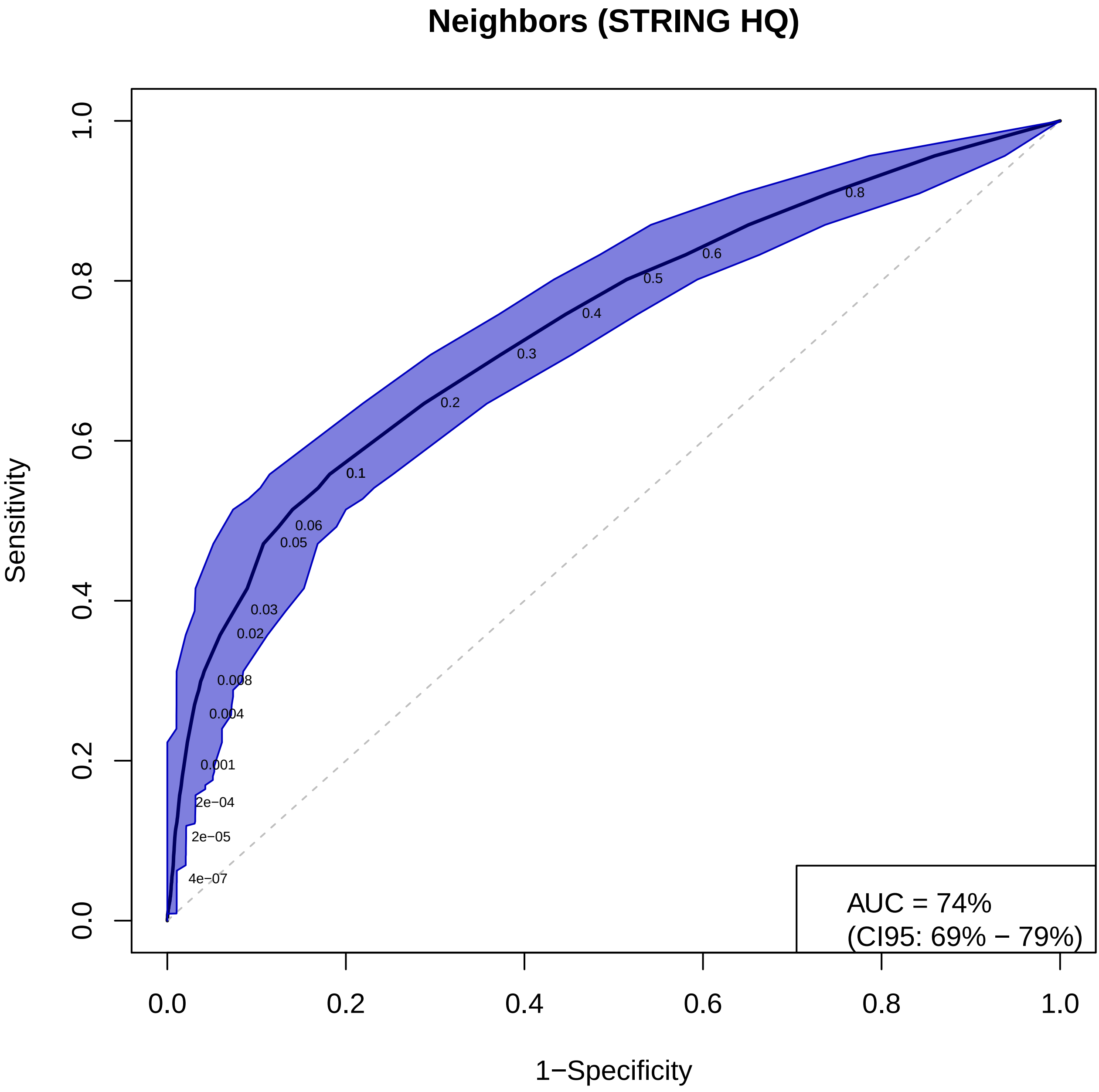

Neighbors (STRING HQ)
Sensitivity
1−Specificity
0.0
0.2
0.4
0.6
0.8
1.0
4e−07
2e−05
2e−04
0.001
0.004
0.008
0.02
0.03
0.05
0.06
0.1
0.2
0.3
0.4
0.5
0.6
0.8
AUC = 74%
(CI95: 69% − 79%)

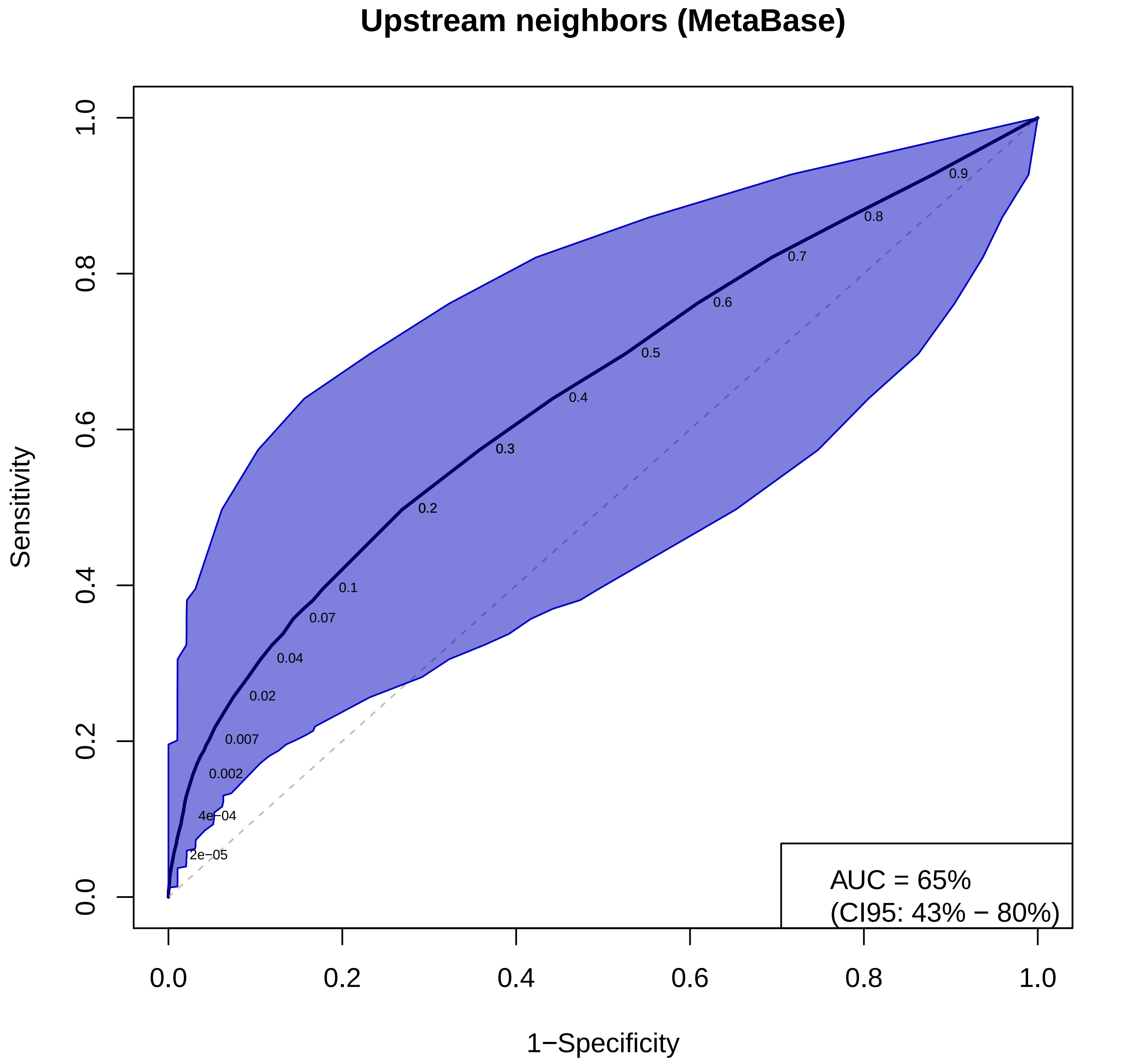

Upstream neighbors (MetaBase)
Sensitivity
1−Specificity
0.0
0.2
0.4
0.6
0.8
1.0
2e−05
4e−04
0.002
0.007
0.02
0.04
0.07
0.1
0.2
0.3
0.4
0.5
0.6
0.7
0.8
0.9
AUC = 65%
(CI95: 43% − 80%)

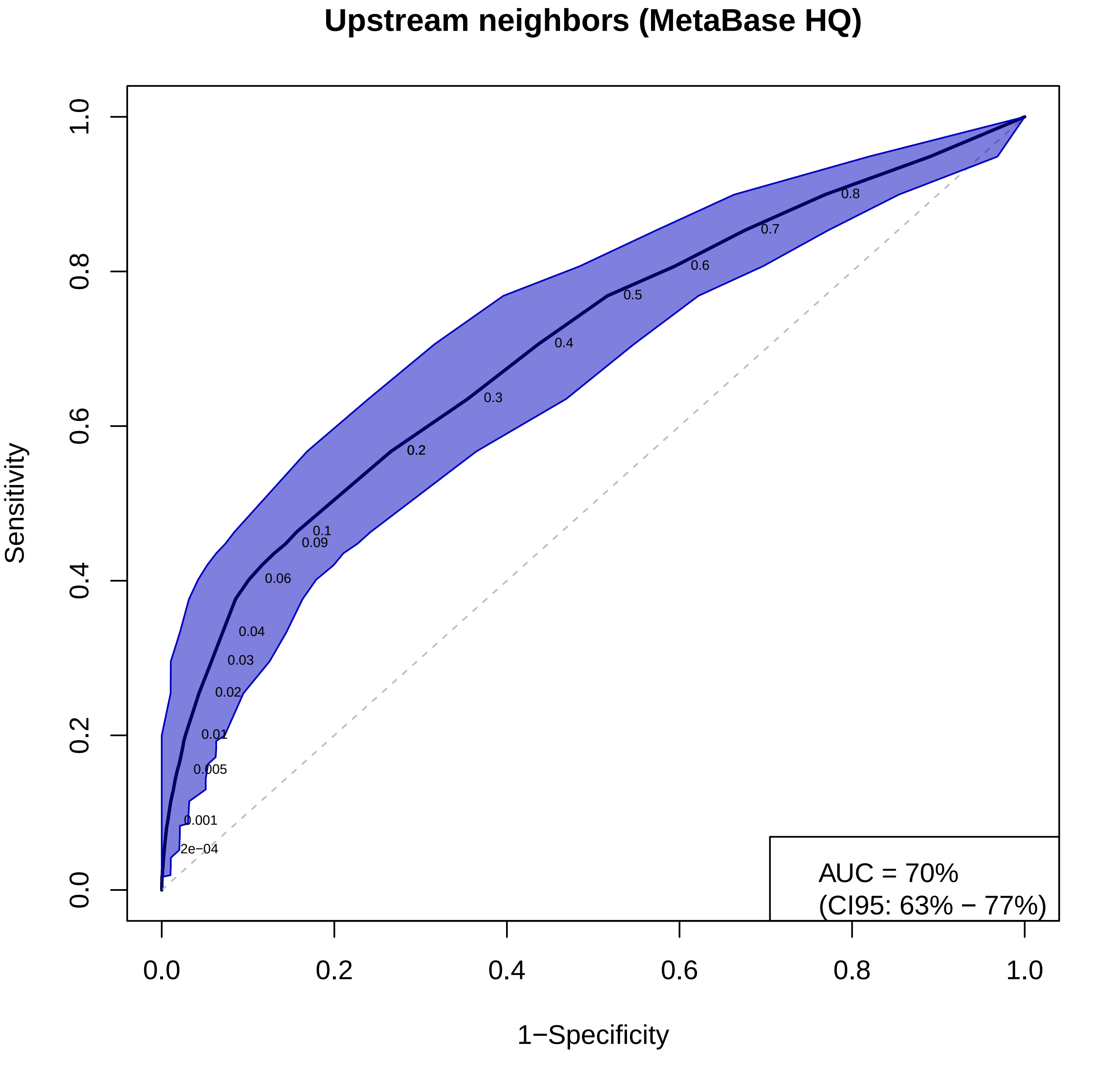

Upstream neighbors (MetaBase HQ)
Sensitivity
1−Specificity
0.0
0.2
0.4
0.6
0.8
1.0
0.8
0.7
0.6
0.5
0.4
0.3
0.2
0.1
0.09
0.06
0.04
0.03
0.02
0.01
0.005
0.001
2e−04
AUC = 70%
(CI95: 63% − 77%)

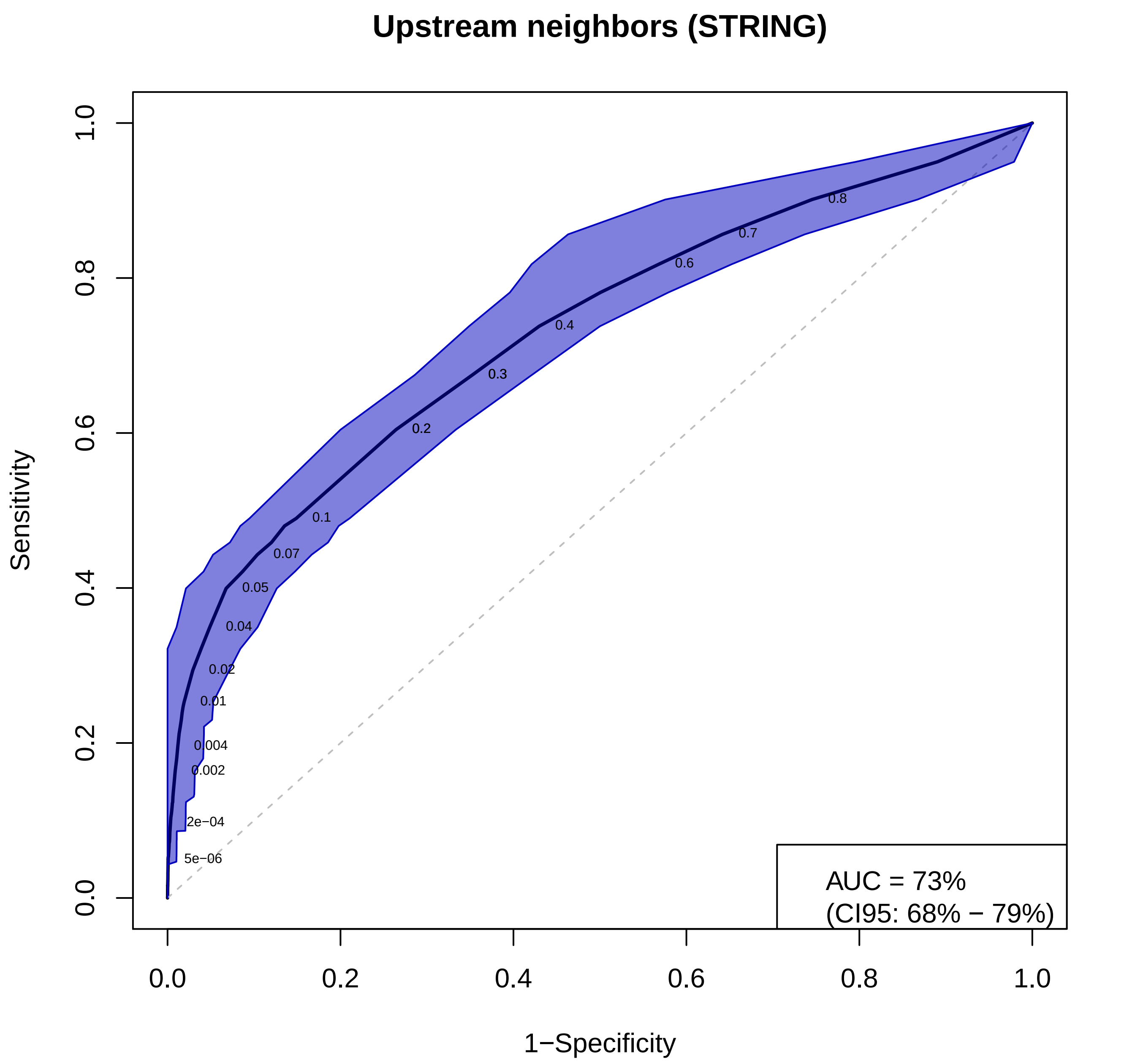

Upstream neighbors (STRING)
Sensitivity
1−Specificity
0.0
0.2
0.4
0.6
0.8
1.0
5e−06
2e−04
0.002
0.004
0.01
0.02
0.04
0.05
0.07
0.1
0.2
0.3
0.4
0.6
0.7
0.8
AUC = 73%
(CI95: 68% − 79%)

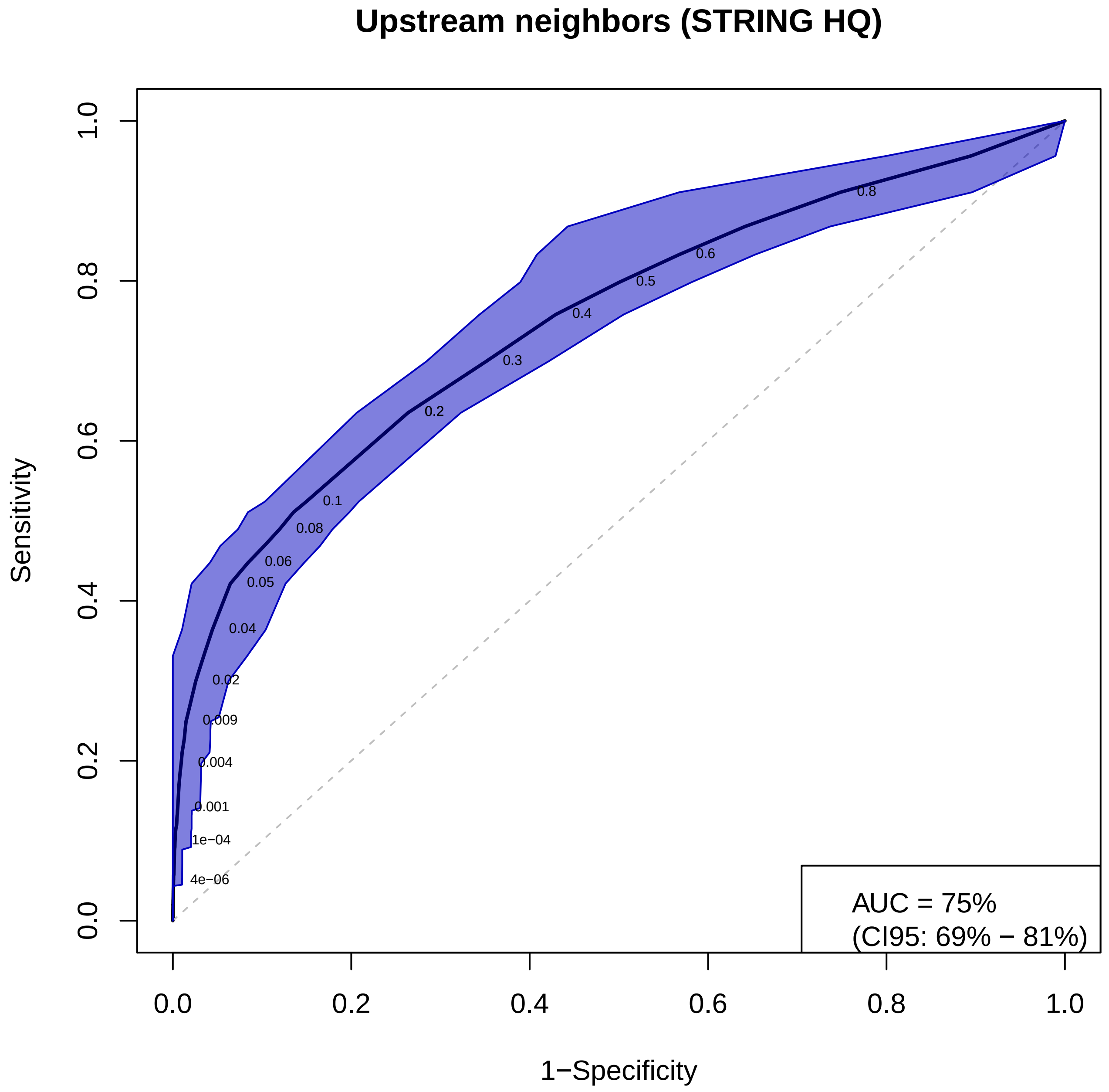

Upstream neighbors (STRING HQ)
Sensitivity
1−Specificity
0.0
0.2
0.4
0.6
0.8
1.0
4e−06
1e−04
0.001
0.004
0.009
0.02
0.04
0.05
0.06
0.08
0.1
0.2
0.3
0.4
0.5
0.6
0.8
AUC = 75%
(CI95: 69% − 81%)

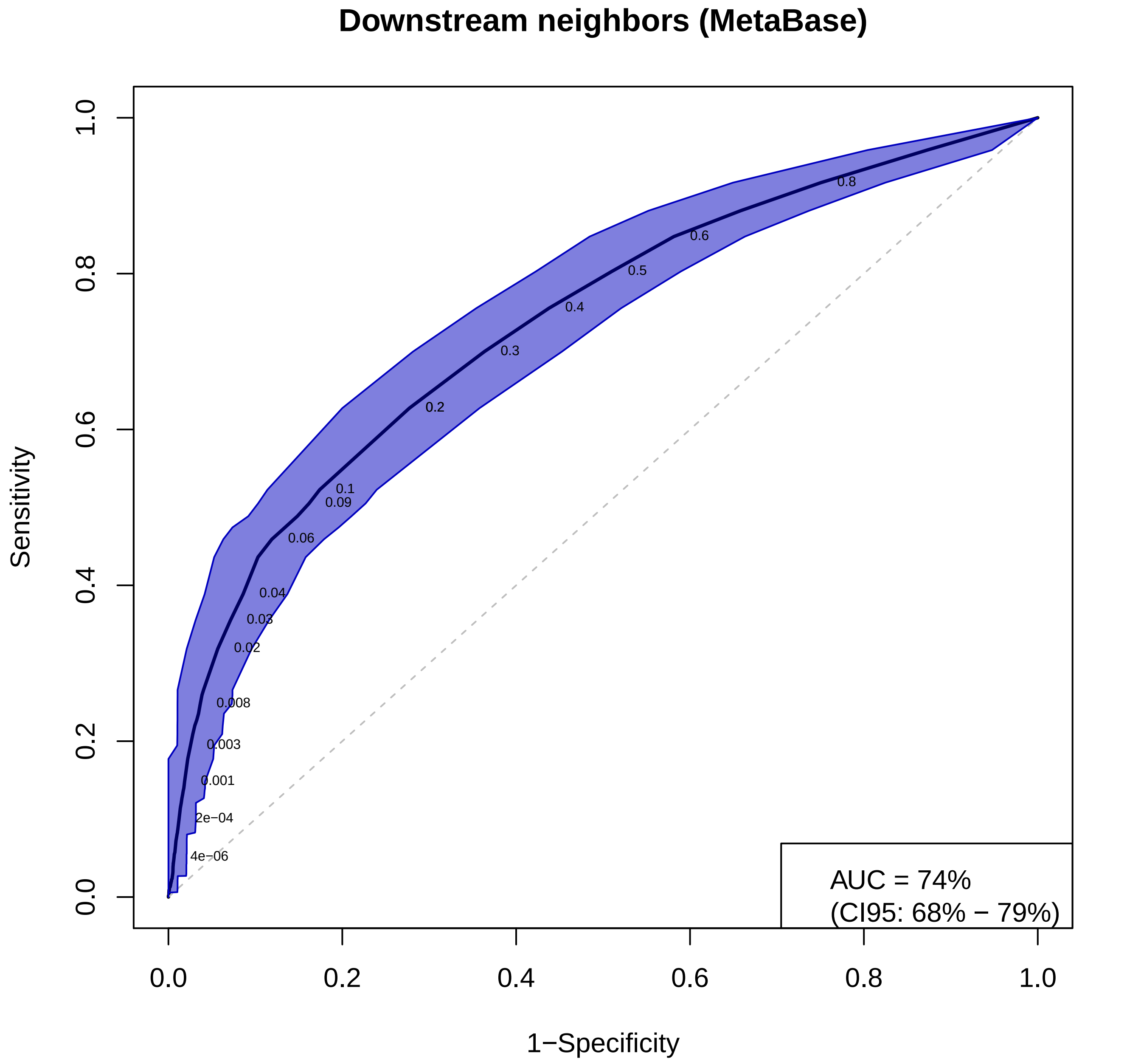

Downstream neighbors (MetaBase)
Sensitivity
1−Specificity
0.0
0.2
0.4
0.6
0.8
1.0
AUC = 74%
(CI95: 68% − 79%)
4e−06
2e−04
0.001
0.003
0.008
0.02
0.03
0.04
0.06
0.09
0.1
0.2
0.3
0.4
0.5
0.6
0.8

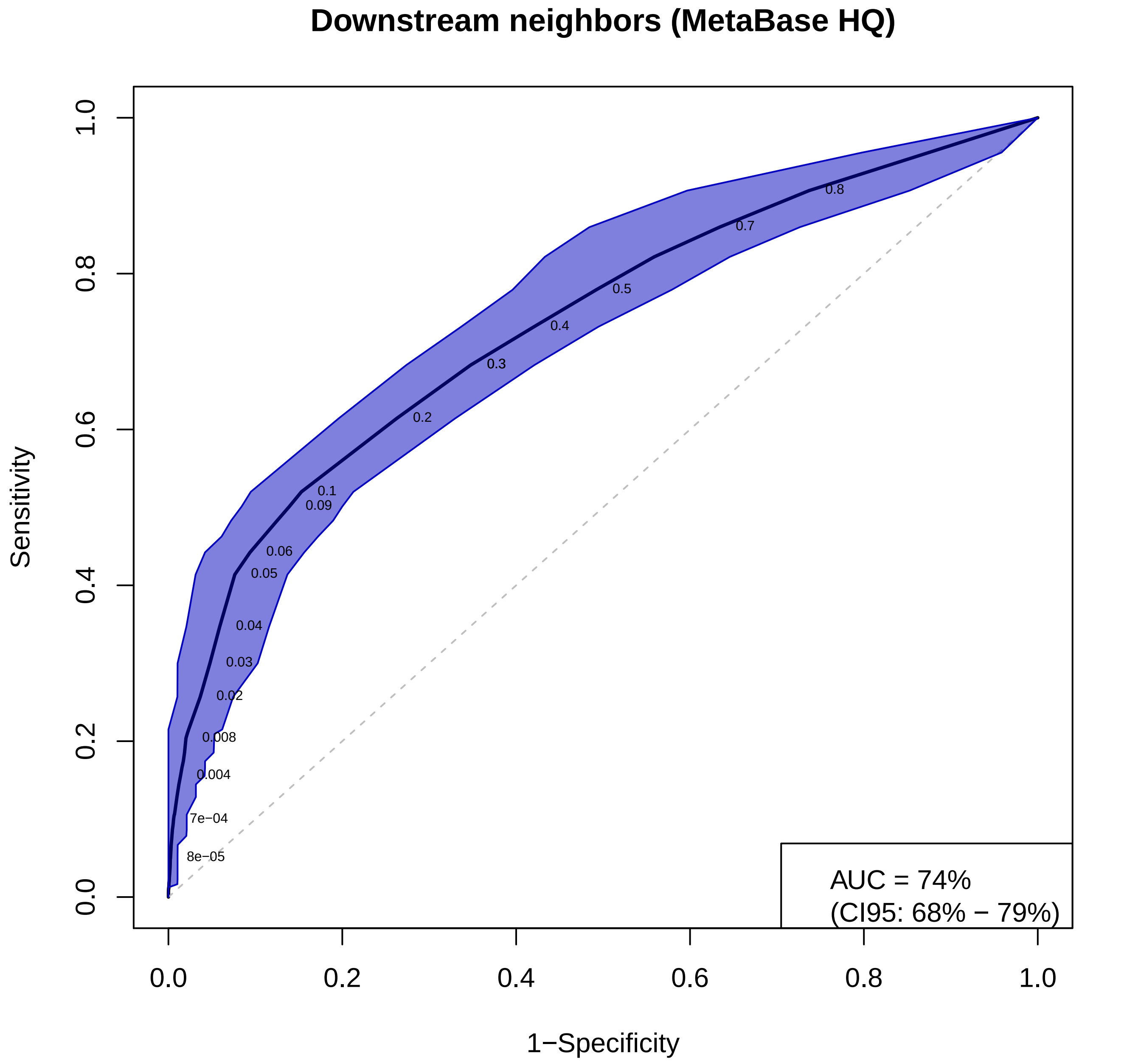

Downstream neighbors (MetaBase HQ)
Sensitivity
1−Specificity
0.0
0.2
0.4
0.6
0.8
1.0
0.0
0.2
0.4
0.6
0.8
1.0
8e−05
7e−04
0.004
0.008
0.02
0.03
0.04
0.05
0.06
0.09
0.1
0.2
0.3
0.4
0.5
0.7
0.8
AUC = 74%
(CI95: 68% − 79%)

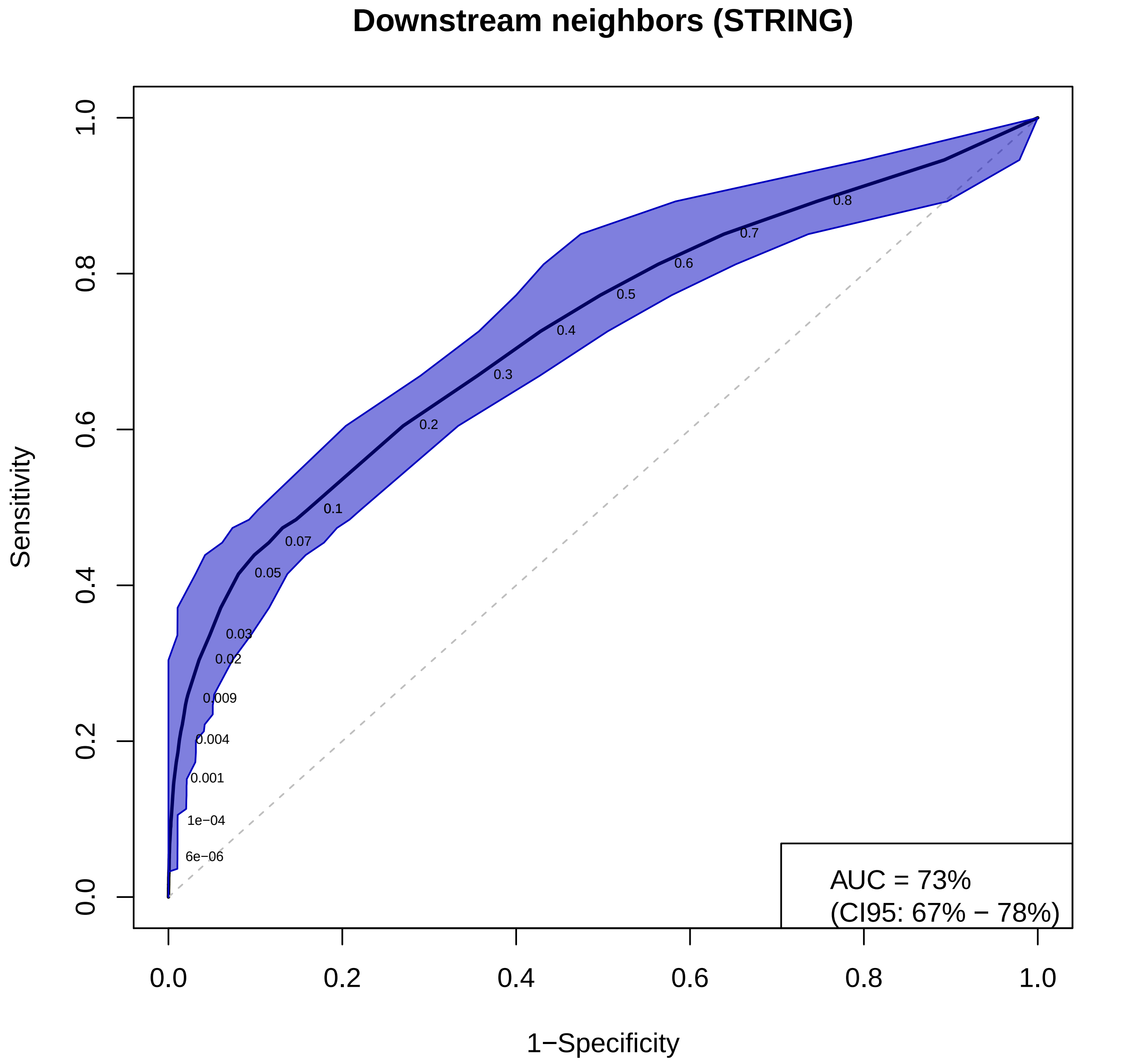

Downstream neighbors (STRING)
Sensitivity
1−Specificity
0.0
0.2
0.4
0.6
0.8
1.0
6e−06
1e−04
0.001
0.004
0.009
0.02
0.03
0.05
0.07
0.1
0.2
0.3
0.4
0.5
0.6
0.7
0.8
AUC = 73%
(CI95: 67% − 78%)

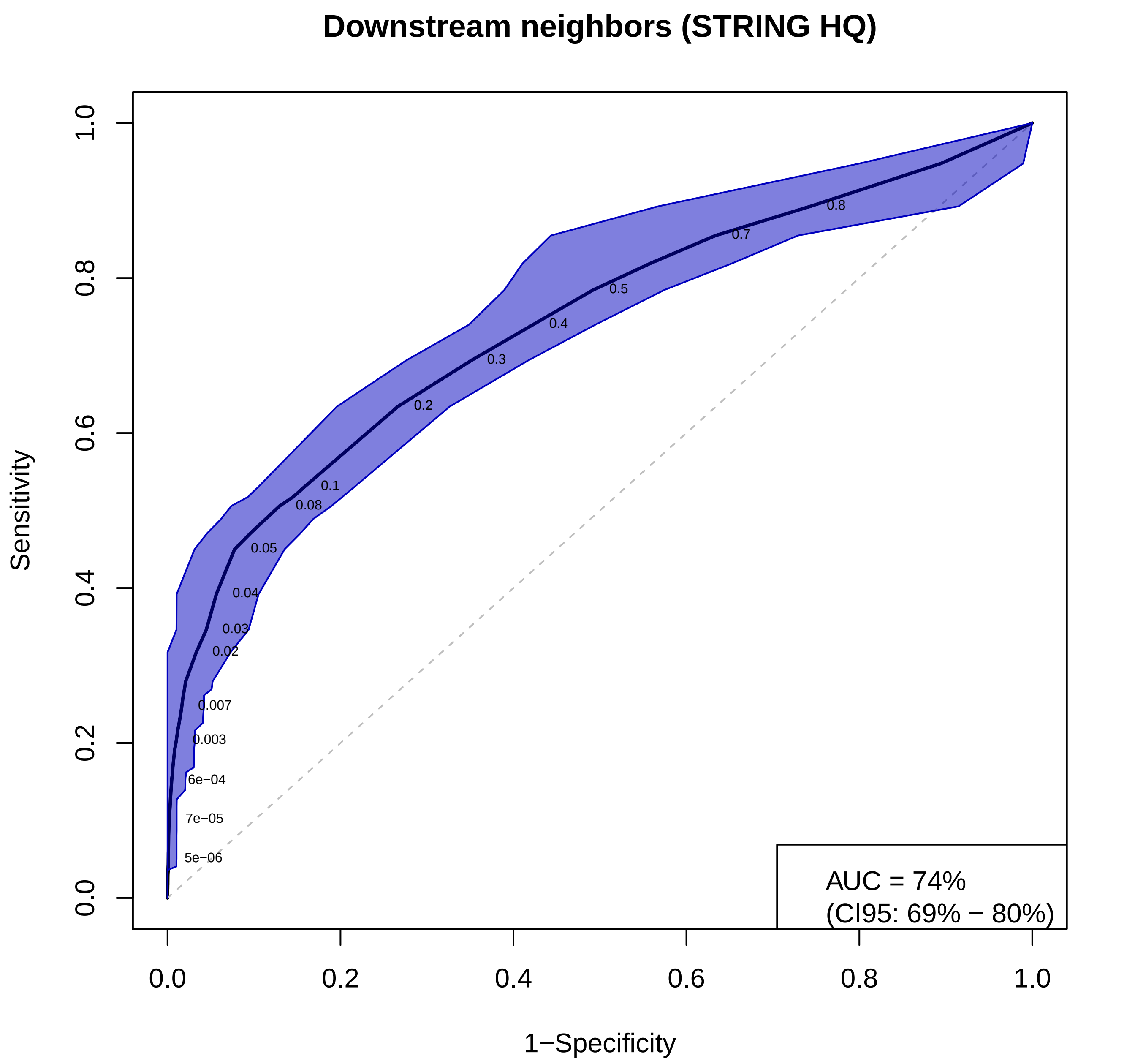

Downstream neighbors (STRING HQ)
Sensitivity
1−Specificity
0.0
0.2
0.4
0.6
0.8
1.0
5e−06
7e−05
6e−04
0.003
0.007
0.02
0.03
0.04
0.05
0.08
0.1
0.2
0.3
0.4
0.5
0.7
0.8
AUC = 74%
(CI95: 69% − 80%)

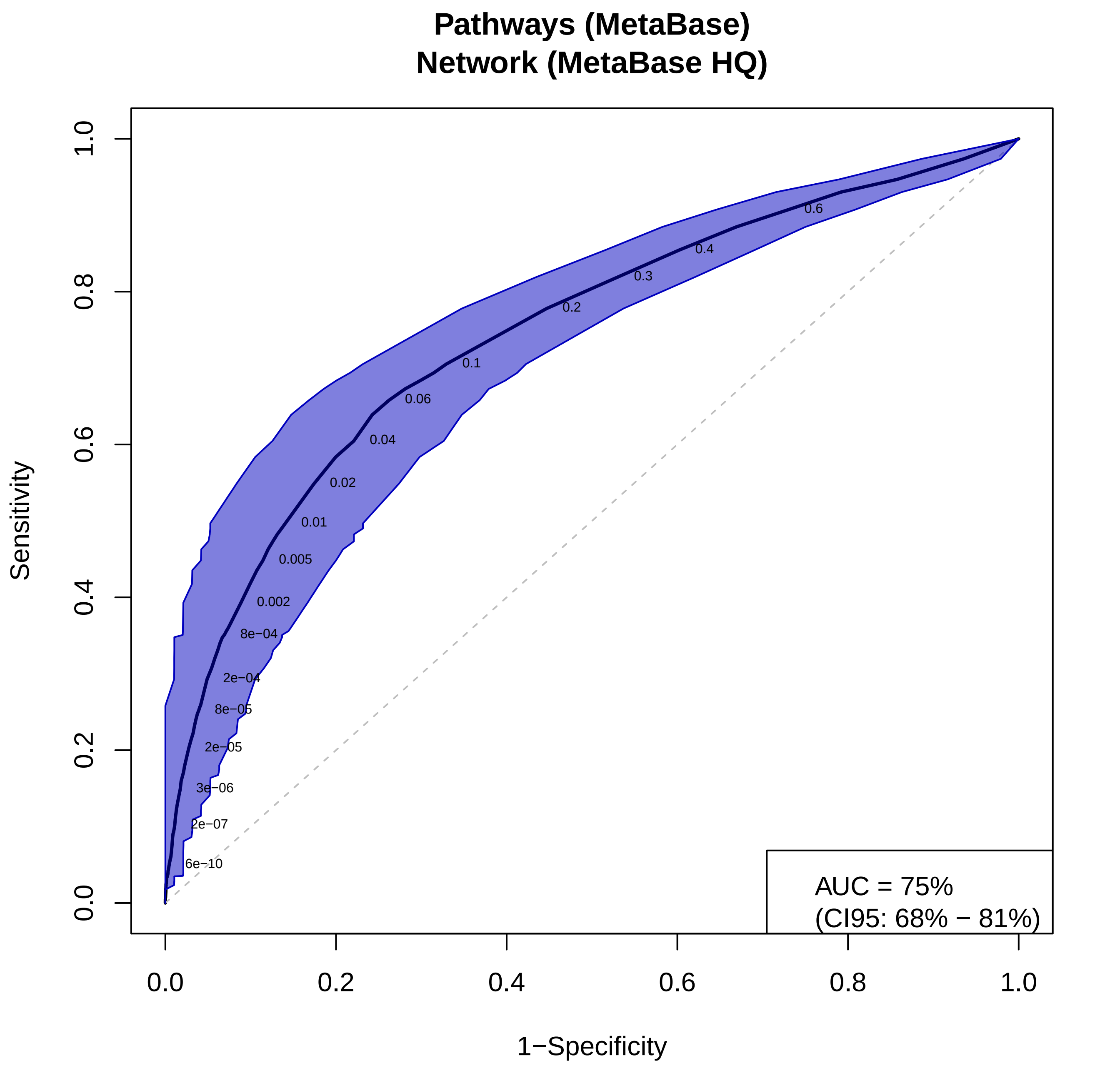

Pathways (MetaBase)
Network (MetaBase HQ)
Sensitivity
1−Specificity
0.0
0.2
0.4
0.6
0.8
1.0
6e−10
2e−07
3e−06
2e−05
8e−05
2e−04
8e−04
0.002
0.005
0.01
0.02
0.04
0.06
0.1
0.2
0.3
0.4
0.6
AUC = 75%
(CI95: 68% − 81%)

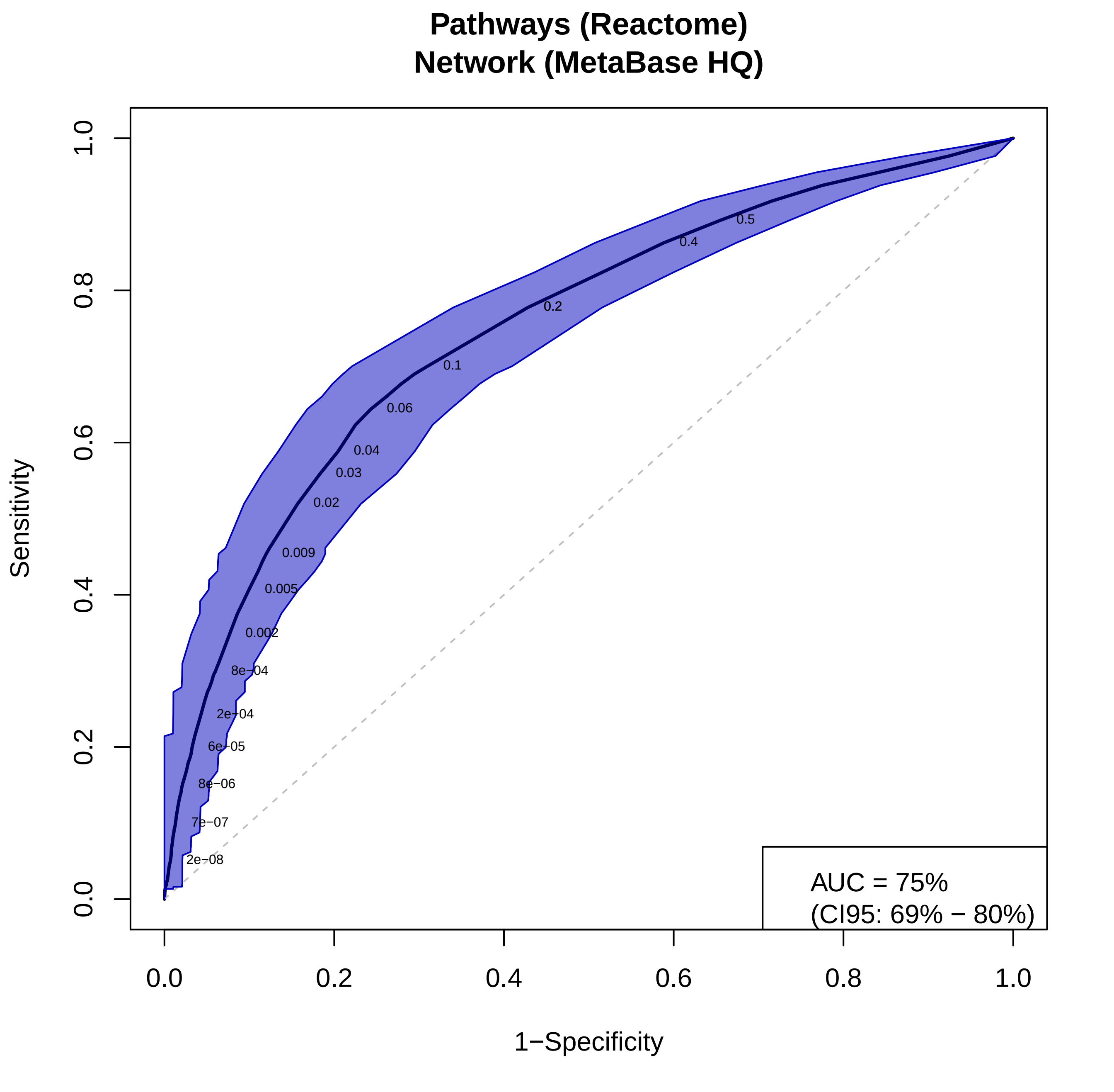
Pathways (Reactome)
Network (MetaBase HQ)
Sensitivity
1−Specificity
0.0
0.2
0.4
0.6
0.8
1.0
2e−08
7e−07
8e−06
6e−05
2e−04
8e−04
0.002
0.005
0.009
0.02
0.03
0.04
0.06
0.1
0.2
0.4
0.5
AUC = 75%
(CI95: 69% − 80%)

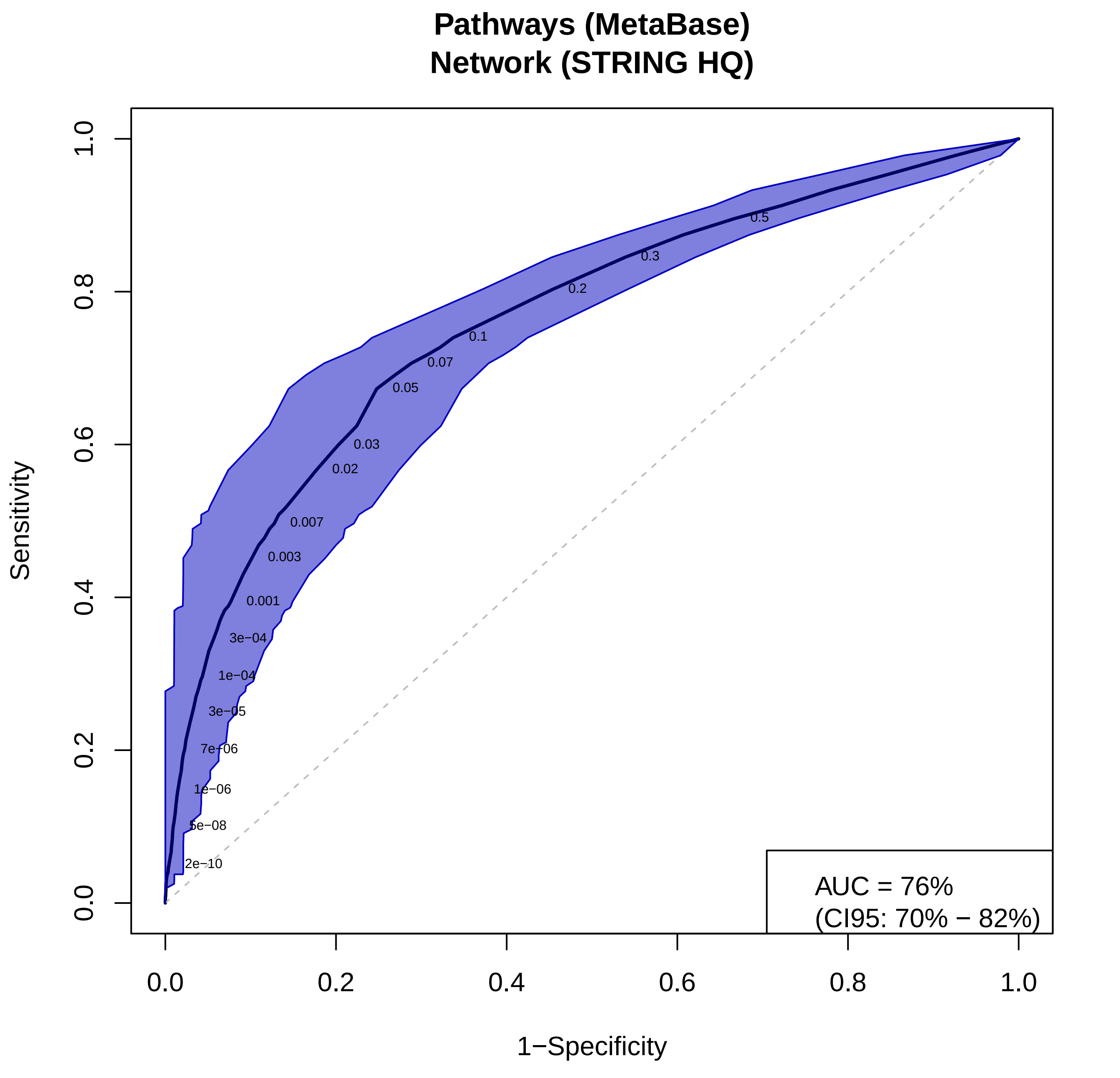

Pathways (MetaBase)
Network (STRING HQ)
Sensitivity
1−Specificity
0.0
0.2
0.4
0.6
0.8
1.0
2e−10
5e−08
1e−06
7e−06
3e−05
1e−04
3e−04
0.001
0.003
0.007
0.02
0.03
0.05
0.07
0.1
0.2
0.3
0.5
AUC = 76%
(CI95: 70% − 82%)

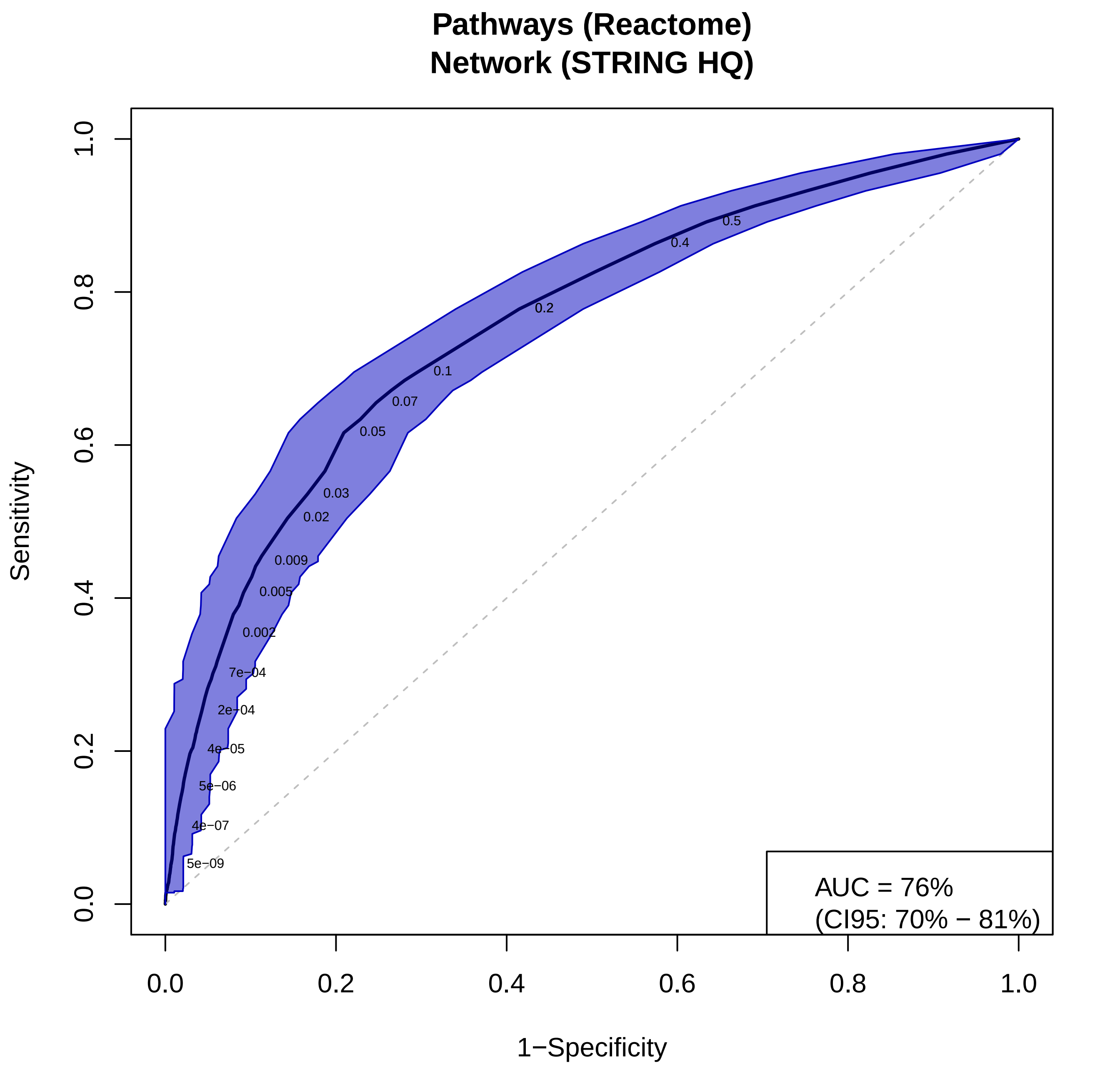

Pathways (Reactome)
Network (STRING HQ)
Sensitivity
1−Specificity
0.0
0.2
0.4
0.6
0.8
1.0
5e−09
4e−07
5e−06
4e−05
2e−04
7e−04
0.002
0.005
0.009
0.02
0.03
0.05
0.07
0.1
0.2
0.4
0.5
AUC = 76%
(CI95: 70% − 81%)
