# Supplementary material for: PCAN: phenotype consensus analysis to support disease-gene association
Source: BMC Bioinformatics. 2016 Dec 7;17:518. doi: 10.1186/s12859-016-1401-2 (PMC5142268; doi:10.1186/s12859-016-1401-2)
Supplement: Additional file 3: Figure S1. — Comparison of genes within the “Anchoring of the basal body to the plasma membrane” pathway to HP terms describing Joubert syndrome. (a) Distribution of symmetric semantic similarity scores of genes for the 8 HP terms related to Joubert syndrome. The red bars correspond to the distribution of the scores of genes belonging to the pathway of interest. The grey bars correspond to the distribution of the scores for all the other genes. (The density of scores equal to 0 is truncated; its actual value is 12.8). (b) Symmetric semantic similarity scores of genes belonging to the pathway of interest. The gene candidate, CC2D2A, is highlighted. In the supplementary figure, the solid red line corresponds to the quantiles of the scores of all the genes. Dashed red lines show the value of three specific quantiles: 50, 75 and 95%. (c) Heatmap showing the best semantic similarity between each gene in the pathway of interest (columns) and each HP term under focus (rows). The red intensity of each square corresponds to the highest semantic similarity score between the HP term of interest and the gene associated HP terms (white: 0 and red: 5.2). The gene candidate, CC2D2A, is highlighted. (PPTX 198 kb) [file 12859_2016_1401_MOESM3_ESM.pptx]

## Slide 1
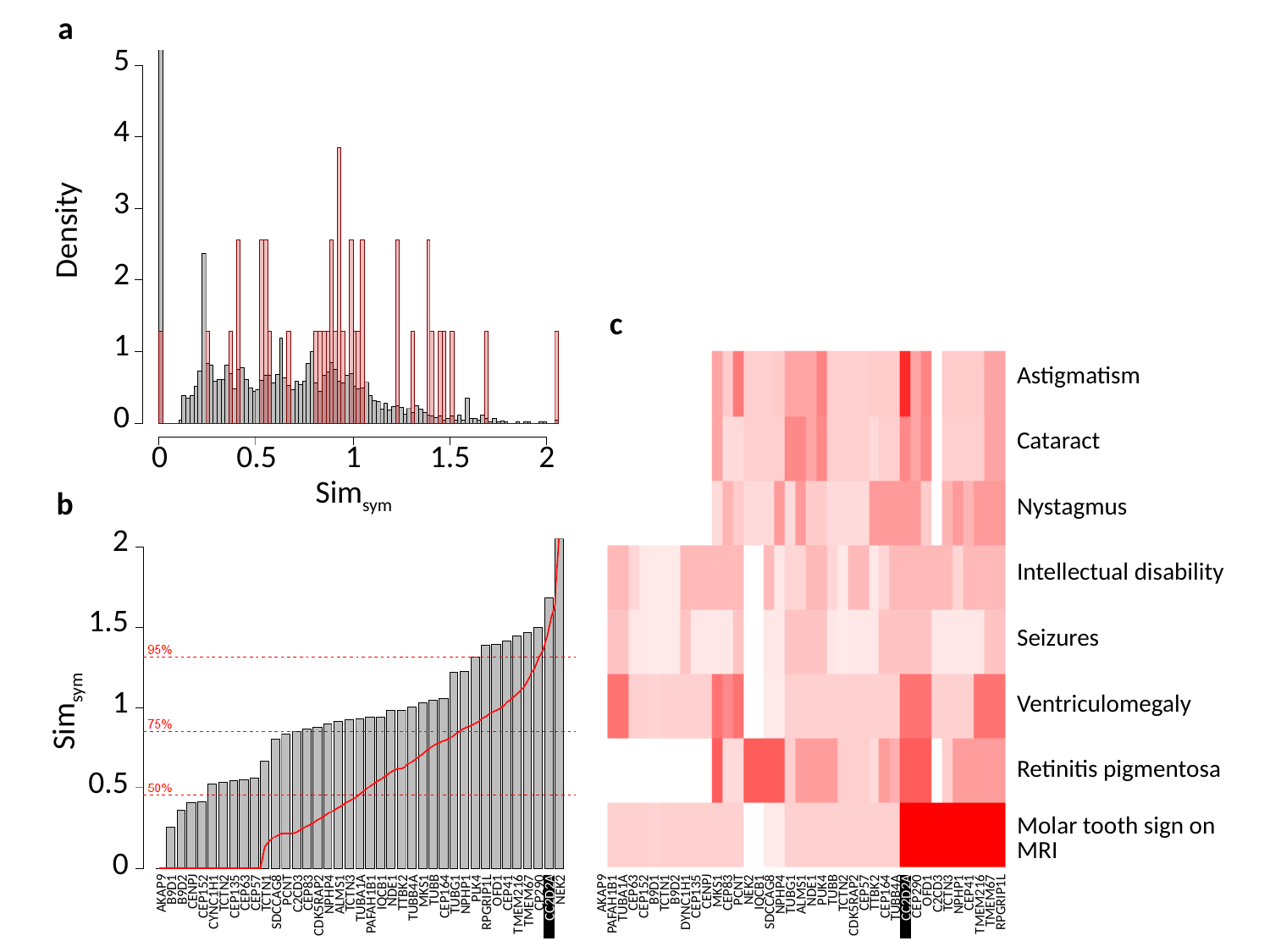

a
| 5 |
| --- |
| 4 |
| 3 |
| 2 |
| 1 |
| 0 |
Density
c
| Astigmatism |
| --- |
| Cataract |
| Nystagmus |
| Intellectual disability |
| Seizures |
| Ventriculomegaly |
| Retinitis pigmentosa |
| Molar tooth sign on MRI |
| 0 | 0.5 | 1 | 1.5 | 2 |
| --- | --- | --- | --- | --- |
Simsym
b
| 2 |
| --- |
| 1.5 |
| 1 |
| 0.5 |
| 0 |
Simsym
| AKAP9 | B9D1 | B9D2 | CENPJ | CEP152 | CYNC1H1 | TCTN2 | CEP135 | CEP63 | CEP57 | TCTN1 | SDCCAG8 | PCNT | C2CD3 | CEP83 | CDK5RAP2 | NPHP4 | ALMS1 | TCTN3 | TUBA1A | PAFAH1B1 | IQCB1 | NDE1 | TTBK2 | TUBB4A | MKS1 | TUBB | CEP164 | TUBG1 | NPHP1 | PLK4 | RPGRIP1L | OFD1 | CEP41 | TMEM216 | TMEM67 | CP290 | CC2D2A | NEK2 |
| --- | --- | --- | --- | --- | --- | --- | --- | --- | --- | --- | --- | --- | --- | --- | --- | --- | --- | --- | --- | --- | --- | --- | --- | --- | --- | --- | --- | --- | --- | --- | --- | --- | --- | --- | --- | --- | --- | --- |
| AKAP9 | PAFAH1B1 | TUBA1A | CEP63 | CEP152 | B9D1 | TCTN1 | B9D2 | DYNC1H1 | CEP135 | CENPJ | MKS1 | CEP83 | PCNT | NEK2 | IQCB1 | SDCCAG8 | NPHP4 | TUBG1 | ALMS1 | NDE1 | PUK4 | TUBB | TCTN2 | CDK5RAP2 | CEP57 | TTBK2 | CEP164 | TUBB4A | CC2D2A | CEP290 | OFD1 | C2CD3 | TCTN3 | NPHP1 | CEP41 | TMEM216 | TMEM67 | RPGRIP1L |
| --- | --- | --- | --- | --- | --- | --- | --- | --- | --- | --- | --- | --- | --- | --- | --- | --- | --- | --- | --- | --- | --- | --- | --- | --- | --- | --- | --- | --- | --- | --- | --- | --- | --- | --- | --- | --- | --- | --- |
